# Spatially Resolved Top-Down Proteomics of Tissue Sections Based on a Microfluidic Nanodroplet Sample Preparation Platform

**DOI:** 10.1016/j.mcpro.2022.100491

**Published:** 2023-01-02

**Authors:** Yen-Chen Liao, James M. Fulcher, David J. Degnan, Sarah M. Williams, Lisa M. Bramer, Dušan Veličković, Kevin J. Zemaitis, Marija Veličković, Ryan L. Sontag, Ronald J. Moore, Ljiljana Paša-Tolić, Ying Zhu, Mowei Zhou

**Affiliations:** 1 Environmental Molecular Sciences Laboratory, Pacific Northwest National Laboratory, Richland, Washington, USA; 2 Biological Sciences Division, Pacific Northwest National Laboratories, Richland, Washington, USA

**Keywords:** top-down proteomics, nanoPOTS, laser capture microdissection, proteoform, spatial proteomics, quantitation, ABC, ammonium bicarbonate, ACN, acetonitrile, BUP, bottom-up proteomics, CV, compensation voltages, DDM, n-dodecyl-beta-maltoside, DMSO, dimethyl sulfoxide, FA, formic acid, FAIMS, field asymmetric ion mobility spectrometry, HEK293, human embryonic kidney 293 cell line, LCM, Laser Capture Microdissection, LC-MS/MS, liquid chromatography with tandem mass spectrometry, MSI, mass spectrometry imaging, nanoPOTS, nanodroplet Processing in One pot for Trace Samples, PfCs, proteoform clusters, PP, polypropylene, PPI, protein–protein interaction, ProMex, Protein Mass Extractor, PrSMs, proteoform spectrum matches, PTM, posttranslational modification, RT, retention time, STRING, Search Tool for the Retrieval of Interacting Gene/Proteins, TCEP, Tris(2-carboxyethyl)phosphine, TDP, top-down proteomics, TFA, tri-fluoroacetic acid, TopFD, Top-down mass spectrometry feature detection, TopPIC, Top-down mass spectrometry-based Proteoform Identification and Characterization

## Abstract

Conventional proteomic approaches measure the averaged signal from mixed cell populations or bulk tissues, leading to the dilution of signals arising from subpopulations of cells that might serve as important biomarkers. Recent developments in bottom-up proteomics have enabled spatial mapping of cellular heterogeneity in tissue microenvironments. However, bottom-up proteomics cannot unambiguously define and quantify proteoforms, which are intact (*i.e.*, functional) forms of proteins capturing genetic variations, alternatively spliced transcripts and posttranslational modifications. Herein, we described a spatially resolved top-down proteomics (TDP) platform for proteoform identification and quantitation directly from tissue sections. The spatial TDP platform consisted of a nanodroplet processing in one pot for trace samples–based sample preparation system and an laser capture microdissection–based cell isolation system. We improved the nanodroplet processing in one pot for trace samples sample preparation by adding benzonase in the extraction buffer to enhance the coverage of nucleus proteins. Using ∼200 cultured cells as test samples, this approach increased total proteoform identifications from 493 to 700; with newly identified proteoforms primarily corresponding to nuclear proteins. To demonstrate the spatial TDP platform in tissue samples, we analyzed laser capture microdissection–isolated tissue voxels from rat brain cortex and hypothalamus regions. We quantified 509 proteoforms within the union of top-down mass spectrometry–based proteoform identification and characterization and TDPortal identifications to match with features from protein mass extractor. Several proteoforms corresponding to the same gene exhibited mixed abundance profiles between two tissue regions, suggesting potential posttranslational modification–specific spatial distributions. The spatial TDP workflow has prospects for biomarker discovery at proteoform level from small tissue sections.

## Highlights

• Top-down proteomics of small tissue sections was demonstrated.
• Proteoforms specific to anatomical regions in rat brain were detected.
• An integrated informatics workflow for proteoform quantitation was presented.

## Graphical Abstract

**Figure:**
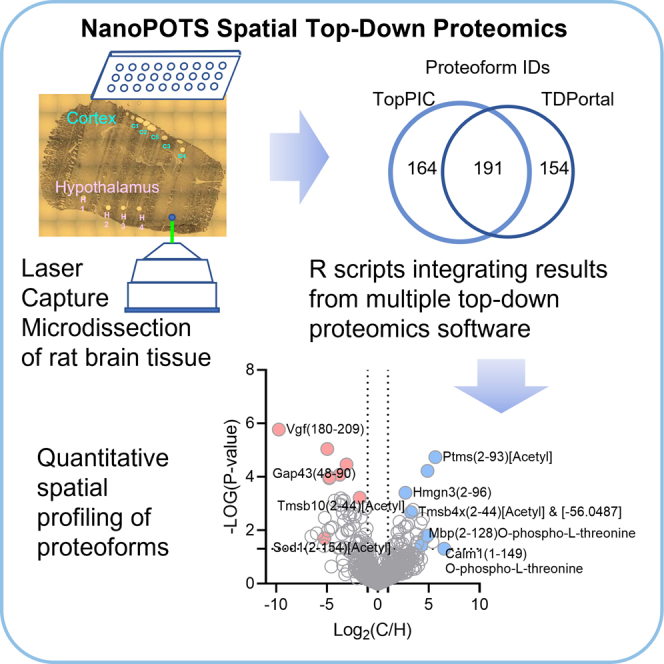

## In Brief

We demonstrated a spatial top-down proteomics (TDP) workflow for small rat brain tissue sections corresponding to ∼200 cells from distinct anatomical regions for understanding the cellular heterogeneity. The experimental protocol used a laser capture microdissection—nanoPOTS platform for liquid chromatography mass spectrometry of proteoforms extracted from tissue. Nine spots were analyzed using an informatics pipeline integrating multiple TDP software, yielding 509 quantifiable proteoforms. Distinct changes of proteoforms not easily accessible from protein-level measurements could reveal new mechanisms for biomarker discovery.

Top-down proteomics (TDP) is a mass spectrometry (MS) strategy for characterizing “proteoforms”, which encompass the combination of posttranslational modifications (PTMs), splice-isoforms, and amino acid variants occurring on a protein sequence (1). These variations at the proteoform level are not directly encoded in the genes. Still, they are critical to regulating cellular functions, particularly in the case of histones where co-occurrence of PTMs is known to influence chromatin biology and epigenetic regulation of genes (2). Combinatorial PTMs present a significant challenge for bottom-up proteomics (BUP) or antibody-based methods (3, 4, 5). TDP avoids ambiguity associated with proteoform inference from peptides by bypassing proteolytic steps (6, 7). Achieving high-quality proteoform identification with TDP, however, is challenging as it needs sufficient protein sample amount, high MS performance, and efficient fragmentation for confident assignment of PTMs. Thus, TDP typically requires bulk-scale tissue or large quantities of cultured cells (∼10^6^) to obtain sufficient proteoform coverages. Encouragingly, recent developments in MS instrumentation, methods, and informatics have significantly improved attainable sensitivity and depth of coverage (8, 9, 10, 11, 12) and thus allowed for reduced sample requirement toward single-cell levels (13, 14). These advances have enabled the characterization of cellular heterogeneity among isolated cell populations or tissue regions (*e.g*., functional tissue units) that contain specific morphological and functional biomarkers (15, 16, 17). However, most of these advances were made for BUP analysis, obscuring the critical information needed for proteoform characterization.

Several microsampling and MS detection methods have been developed to enable highly sensitive and spatially resolved TDP analysis. Most of these advances were achieved employing MS imaging (MSI) methods, including matrix-assisted laser desorption ionization (MALDI) (18), nanospray desorption electrospray ionization (19, 20), liquid extraction surface analysis (21), and laser ablation electrospray ionization (22). However, directly identifying proteins with MS/MS fragmentation in MSI is not trivial due to overlapping signals, salt adducts, and low signal intensity (23). In MALDI MSI, an extra challenge is that ions typically have low charge states (≤3), which greatly reduces fragmentation efficiency (18). For this reason, intact protein databases or prior knowledge from MS profiles and fragmentation patterns are required for peak assignment (18). Additionally, because of the lack of separation, MSI methods are typically limited to detecting highly abundant or highly ionizable proteins. To address these challenges, liquid microjunction microextraction (24), parafilm-assisted microdissection (24), and laser capture microdissection (LCM) (15) have been explored to isolate and characterize microstructures from tissue sections for more in-depth characterization. For example, the integration of LCM and capillary electrophoresis with TDP has enabled identification of over 400 proteoforms from two different regions of zebrafish brain (15).

Herein, we describe an improved spatial TDP platform that integrates LCM-based sample isolation with our previously developed nanodroplet processing in one-pot for trace samples (nanoPOTS) sample preparation. We have demonstrated that nanoPOTS-based TDP can significantly improve the recovery of low amounts of samples by minimizing protein absorption on container surfaces (25). Over 150 proteoforms were identified from ∼70 cultured HeLa cells, and a variety of PTMs and proteoforms assigned (25). In this work, we further improved the nanoPOTS protocol for enhanced proteoform coverage and extended the application from cultured cells to tissue sections. We added the nuclease benzonase in the extraction buffer to reduce sample viscosity and improve protein extraction efficiency as reported previously for bulk analyses (26, 27). To achieve deeper proteome coverage and more confident identifications, we developed several scripts (available at https://github.com/PNNL-HubMAP-Proteoform-Suite/spatially-resolved-TDP) that integrate qualitative and quantitative results from protein mass extractor (ProMex), top-down mass spectrometry–based proteoform identification and characterization (TopPIC), and TDPortal. To demonstrate the spatial TDP analysis, we employed LCM to isolate cells from the cortex and hypothalamus regions in a rat brain section and detected differential proteoform profiles between the two regions. We found varying proteoform abundance profiles for the same protein (gene), highlighting the need for proteoform-centric measurements. Finally, we demonstrated the identified proteoforms from the LCM-nanoPOTS–TDP analyses can serve as a library to annotate intact protein peaks in MALDI-MSI spectrum. The workflow can be a valuable resource for spatial TDP of tissue sections for biomarker discovery at the proteoform level.

## Experimental Procedures

### Reagents and Chemicals

Deionized water (18.2 MΩ) was purified using a Barnstead Nanopure Infinity system. Tris(2-carboxyethyl)phosphine (TCEP), n-dodecyl-beta-maltoside (DDM) detergent, and protease/phosphatase inhibitor cocktails (catalog 78,430) were purchased from ThermoFisher Scientific. Benzonase nuclease was purchased from EMD Millipore. Magnesium chloride (MgCl_2_), formic acid (FA), 1× phosphate buffer saline, dimethyl sulfoxide (DMSO), tri-fluoroacetic acid (TFA), ethanol (EtOH), FA, and ammonium bicarbonate (ABC) were purchased from Sigma-Aldrich.

### Cell Culture

Human embryonic kidney 293 (HEK293) cells were cultured under Dulbecco's modified Eagle's medium with 10% fetal bovine serum and 1% penicillin streptomycin at 37 °C and 5% CO_2_ atmosphere.

### Rat Brain Tissue Sectioning

Frozen female rat brain, purchased from BioIVT, was mounted on cryomicrotome chuck and then sectioned (10 μm thickness; CryoStar NX70, Thermo Fisher) using temperature of −18 °C and −20 °C, for specimen and blade, respectively. Sections were thaw-mounted onto indium tin oxide–coated glass slides (Bruker Daltonics) for MALDI analysis and onto polyethylene naphthalate membrane slides (Carl Zeiss Microscopy) for LCM coupled to nanoPOTS experiments.

### MALDI Analysis

Samples were vacuum desiccated for 30 min and then washed in fresh solutions of 70% ethanol for 30 s, 100% ethanol for 30 s, Carnoy’s solution (6:3:1 v/v ethanol/chloroform/glacial acetic acid) for 2 min, 100% ethanol for 30 s, water with 0.2% TFA for 15 s, and 100% ethanol for 30 s. Samples were then dried by a stream of nitrogen gas prior to MALDI matrix application. HTX Technologies M5 Sprayer was used to deposit sonicated supernatant of 15 mg/ml 2,5-DHA (2,5-dihydroxyacetophenone) in 90% acetonitrile with 0.2% TFA. The flow rate of the matrix was 150 μl/min with a nozzle temperature of 30.0 °C, with a velocity set to 1300 mm/min with 10 PSI of nitrogen gas. The matrix was then recrystallized with 5% acetic acid solution in water at 38.5 °C and dried for 3.5 min and then immediately analyzed using an elevated pressure MALDI source (Spectroglyph LLC) coupled to a Thermo Scientific Q Exactive HF Orbitrap MS upgraded with ultra-high mass range boards (28). Spectra were acquired over the *m/z* range of 3500 to 20,000 in positive polarity mode with a resolving power of 240k at *m/z* 200 (512 ms transient) and 250 laser shots per pixel. Scans in the.RAW file were summed as a single spectrum for proteoform assignment by accurate mass.

### LCM-nanoPOTS-TDP Sample Preparation

NanoPOTS chips were fabricated on glass substrates using photolithography, followed by a wetting etching solution containing 1 M HF, 0.5 M NH_4_F, and 0.75 M HNO_3_ processed with procedures as described previously (12). Polypropylene (PP) chips were produced by an injection molding company (Proto Labs). Glass or PP chips with an array of 4 × 12 nanowells were used throughout the study. Cells were collected in 1× phosphate buffer saline with protease and phosphatase inhibitor. After cell deposition, 100-nL lysis buffer containing 2 mM MgCl_2_, 10 mM TCEP, and 16 M urea with 0.4% DDM in 50 mM ABC was added into each well, followed by 1-h incubation under room temperature. Next, 200 nl of 2 mM MgCl_2_ with 2.5 unit/μl of benzonase nuclease was added in each well and incubated at 37 °C for 1 h. Finally, the sample was acidified by adding 50 nl of 5% FA into each well and dried in a vacuum chamber.

For tissue samples, the sections were fixed in 70% EtOH for 1 min and dehydrated in 95% and 100% EtOH (1 min per wash). A PALM MicroBeam system (Carl Zeiss MicroImaging) was used to perform cell isolation from different regions of rat brain. For each replicate, tissue voxels with an area of 100,000 μm^2^ were excised and collected in PP microPOTS chip (same design as nanoPOTS chips, but with larger size well of 2.2 mm diameter instead of 1.2 mm) preloaded with 2 μl DMSO as capture liquid. Before protein extraction, DMSO was evaporated by heating the chip to 70 °C. Next, we added 2 μl lysis buffer in each well that contained 2.5 unit/μl benzonase nuclease, 2 mM MgCl_2_, 10 mM TCEP, 0.2% DDM, and 4M urea in 50 mM ABC, followed by 1-h incubation at 37 °C. The sample was acidified by adding 500 nl of 5% FA into each well and dried in a vacuum chamber. Dried microPOTS chips were frozen at −20 °C or directly used for LC-MS/MS analyses.

### LC-MS/MS Analysis

SPE columns (150 μm i.d.,4 cm long) and the analytical columns (100 μm i.d., 50 cm long) were packed in-house using C2 particles (SMTC2MEB2-3-300) from Separation Methods Technologies. A home-built autosampler system was used for direct sample injection from nanoPOTS chip (29). The injected samples were loaded and desalted on SPE column by infusing with 0.1% FA at 3 μl/min for 5 min. A Dionex nanoUPLC pump (NCP-3200RS, ThermoScientific) system was used with 0.1% FA in H_2_O (buffer A) and 0.1% FA in acetonitrile (buffer B). The LC gradient was programmed as a 120 min gradient from 10% to 50% buffer B followed by a 5 min linear gradient to 80% solvent B. The column was then washed with 70% solvent B for 5 min and re-equilibrated with 5% solvent B for 15 min. The LC flow rates were set at 300 nl/min for the 100-μm column.

Data were collected using Orbitrap Lumos Tribrid and Eclipse mass spectrometers (Thermo Scientific) in data-dependent acquisition mode. We applied field asymmetric ion mobility spectrometry (FAIMS) with compensation voltages (CVs) of −30 V, −40 V, and −50 V (30) to improve signal-to-noise ratio and enhance proteoform coverage (31, 32). Precursor ion mass spectra were acquired with a resolution of 120,000 (at m/z 200), a maximum injection time of 250 ms, a scan range of 600 < *m*/*z* < 2000, an AGC target of 5E5, and five microscans. Precursor ions with charges 5+ or higher and intensities above 2E4 were isolated using an isolation window of 2 *m*/*z* for MS/MS analysis. A single charge state was selected for each neutral mass (*i.e.*, proteoform) within 120 s dynamic exclusion. Tandem mass spectra were acquired with a resolution of 120K (at *m/z* = 200), using higher-energy collisional dissociation with stepped collision energy levels (20%, 30%, and 40%), an AGC target of 1× 10^6^, and a maximum injection time with 500 ms. MS raw data and search results were uploaded to MassIVE with accession MSV000089163.

### Proteoform Identification and Quantitation

The FAIMS datasets were separated into individual raw files by FreeStyle (Thermo Scientific) for each CV. All files were deconvoluted with TOP-down mass spectrometry feature detection (33) and searched by TopPIC (34) (ver. 1.4.13). All spectra were processed with the following parameters: mass error tolerance of 15 ppm, only one unexpected modification, proteoform error tolerance with 3.2 Da (for merging proteoforms with similar masses), and combined target and decoy search with an FDR (false discovery rate) threshold of 1%. MS/MS spectra were searched against UniProtKB/Swiss-Prot rat database (downloaded on August, 2021, containing 8131 reviewed, 21,803 TrEMBL, and 1628 VarSplic sequences) or the human database (downloaded on June 29, 2019, containing 20,352 reviewed sequences).

We performed FDR filtering at the protein level, resulting in a global FDR of <1%. To describe ambiguity in proteoform identifications, we implemented a custom R function that determined a proteoform’s “level” of ambiguity, following the five-level classification system (from 1–5 and 1 being unambiguous and 5 being ambiguous in all metrics) defined by the Consortium for TDP (35). Our function accounted for all forms of ambiguity apart from amino acid sequence ambiguity. Open-modification searches, while useful, can sometimes provide erroneous mass shift assignments (36). To address these issues, we performed retention time alignment (LOESS regression) and mass error recalibration for proteoform spectrum matches (PrSMs) using the dataset with the larger number of PrSMs as a reference. Retention times (RTs) were aligned using the apex spectrum (most intense) for each proteoform. Aligned and recalibrated datasets were then clustered using RT and precursor mass for all PrSMs. We refer to these clusters as “proteoform clusters” (PfCs). A minimum of three PrSMs were required per cluster, and PrSMs not meeting this criterion were pooled together as a “noise” cluster and ignored for quantitative analysis. Within each PfC, the proteoform with the highest number of PrSMs was selected to represent the entire cluster. A newer implementation of the workflows for TopPIC post-processing with additional functions are available on GitHub within the R package TopPICR (37). In parallel, we also processed the same data (after splitting CVs) by TDPortal (38) with *Rattus norvegicus* protein dataset (May 2016) and parameters, including high precursor resolution, filter by FDR, and TDPortal’s code set of standard 4.0.0. TDportal adopts a similar approach to the commercial software ProSightPD, which considers all known PTMs and isoforms in the UniProt database for proteoform identification. This is distinct from TopPIC which does not assume preknowledge on PTMs and can provide complementary results. The proteoform identifications were exported as tables using TDViewer for merging with TopPIC results. The script used to accomplish merging of the two search results can be found at https://github.com/PNNL-HubMAP-Proteoform-Suite/spatially-resolved-TDP.

For label-free quantitation of proteoforms, we relied on the feature abundances from ProMex (39) from the InformedProteomics suite. RT alignment of ProMex features was performed with ProMexAlign (39), with each CV separately aligned and missing features replaced with “NA”. We built a custom R script to align the accurate masses and RTs to the feature abundances and proteoform identifications. Redundant proteoforms were first collapsed by PfC in TopPIC results and by accession number and monoisotopic mass in TDPortal results. Only the top-scored (lowest E-value) proteoform was used to represent each unique feature. Next, collapsed TopPIC and TDPortal proteoforms were matched individually to the aligned ProMex tables within 15 ppm *m/z* and ± 4 min mass, and RT tolerances referred to as a “feature group.” We also checked for deisotoping error and merged proteoforms if they fall into the window after shifting its mass by ± 1 and 2 Da.

After concatenating all CVs together, we sorted low-high by mass and assigned a mass group when each subsequent mass was within 1 Da and 15 ppm *m/z* of a previous mass. Within each mass group, we sorted by RT and assigned an RT group when each subsequent RT was within 4 min of the previous RT. Mass and RT groups were then combined to generate a unique “feature group” in which we collapsed all detected features. When two proteoform IDs matched to the same feature group within 4 min elution window, we prioritized IDs without unknown modifications, with TopPIC PfCs not ending with “_0” (the “noise” cluster) and with smaller E-values (supplemental Fig. S1). The initial output from the scripts were further evaluated manually for merging ambiguous features/proteoforms. The final table includes count, max monoisotopic masses, mean RTs, and median intensities, along with TopPIC and TDPortal proteoform annotations. The features were annotated with proteoforms and filtered for downstream analyses, where each proteoform had to be identified in at least two samples. The proteoform abundances were normalized to the median of each sample (combined FAIMS CV), missing values were imputed randomly from a normal distribution with 0.3 widths and downshift 1.8 standard deviations of each sample’s log_2_ intensity distribution by Perseus v.1.6.2.3 (40) and an unpaired *t* test for determining abundance difference between cortex and hypothalamus.

### Pathway and Network Analysis

Protein association networks for the identified proteins were analyzed by STRING database (version 11.5) (41) for high-confidence (score > 0.7) and medium-confidence (0.4 < score < 0.7) protein–protein interaction (PPI) networks. Functional enrichment analysis was performed by ClueGO plugin (version 2.5.8) (42) in Cytoscape (version 3.8.2) (43) against the gene ontology (44), tissue expression database (45), and Kyoto encyclopedia of genes and genomes database (46, 47) using rat (*R. norvegicus*) proteins.

### Experimental Design and Statistical Rationale

To compare the improvement of benzonase treatment, we identified proteoforms from ∼100 HEK293 cells with and without benzonase treatment (n = 5 each) after LC-MS/MS analysis. We depict a scatter plot with cell numbers *versus* identified proteoforms for performing the slope differences after benzonase treatment.

We applied the benzonase treatment to rat brain LCM tissue TDP analysis. We collected five spots from the rat cortex region and four from regions near the hypothalamus. After protein extraction and LC-MS/MS analysis, we used principal component analysis (PCA) to distinguish the protein characteristic from profile of proteoform abundance in each LCM section. PCA was performed by Perseus (40). We also performed PCA for nonimputed data with projection pursuit (48, 49). Plots were created using by GraphPad Prism 9 (GraphPad Software) and R.

## Results

### Benzonase Treatment Improved Proteoform Identifications

**Fig. 1:**
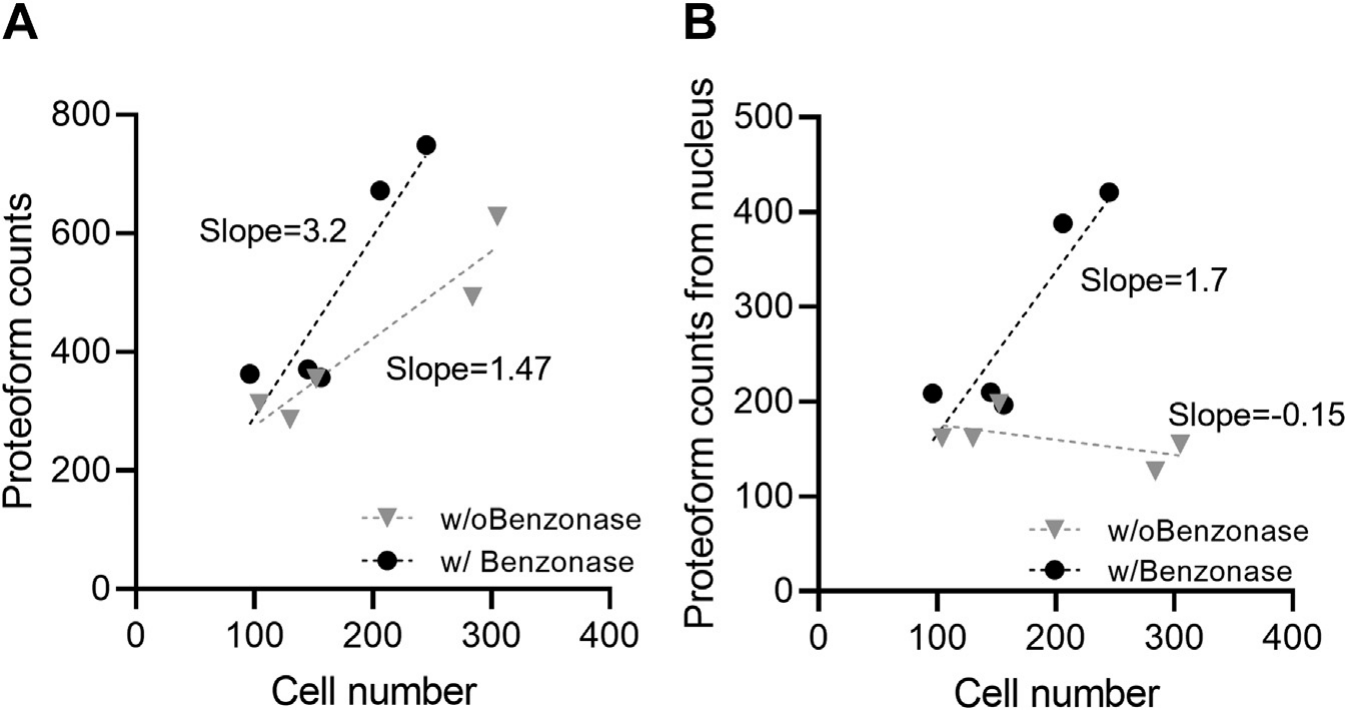
**Proteoform counts with and without benzonase treatment.** Benzonase treatment enhanced both total (*A*) and nucleus (*B*) proteoform identifications at high cell counts. The scatter plots show the relationship of cell number to the number of identified proteoforms with benzonase (*black dots*) and without benzonase (*gray triangles*) treatment, where each point represents one experiment (n = 5 for each condition).

One of the main challenges with TDP is the extraction of intact proteins under conditions compatible with downstream analysis. Viscosity caused by DNA reduced protein extraction efficiency and reproducibility during sample handling/transfer. To address this, we evaluated the effect of benzonase, which has been shown to improve the recovery of nuclear proteins in proteomics preparation (50) by digesting nucleic acid polymers bound to these proteins. The benzonase was added to 100 to 200 HEK293 cells in nanoPOTS wells and analyzed by LC-MS/MS following previous methods (25). Overall, benzonase addition improved nuclear protein recovery at higher cell counts (*p*-value = 0.08) (Fig. 1). We fit linear regression models with the number of identified proteoforms as the response variable and the number of cells as the predictor per sample type (all or nuclear) and treatment type (with or without benzonase) (Fig. 1*A*). At 100 cells or less, the effect of benzonase on proteoform recovery was not significant (*p*-value = 0.2). At cell counts of 165 or greater, proteoform identification were significantly increased. Therefore, sensitivity at this level is likely restricted by LC-MS/MS and not the extraction step.

Based on gene ontology annotation, we separately counted the changes of nuclear proteoforms from total proteoforms. Digestion of DNA strands released more nuclear proteoforms, and benzonase treatment increased proteoforms from cell nucleus significantly (*p*-value = 0.005) (Fig. 1*B*). In addition, we observed the reduced viscosity of sample solution after benzonase treatment, which was consistent with previous reports (50).

We also investigated if the use of PP plastic chip could reduce nonspecific binding–related protein losses. Our previous evaluation indicated PP surface can improve the recovery of peptide samples (51). As shown in supplemental Fig. S2, we found the PP chips yielded a modest increase in the number of identified proteoforms using ∼100 HEK cells as a test sample. With our optimized methodology, we implemented these improvements into our nanoPOTS protocol and applied them to small-scale tissue samples, which represent a more challenging test for protein extractions.

### LCM-NanoPOTS-TDP Enabled the Quantitation of 509 Proteoforms From Two Rat Brain Regions With an Area of ∼100,000 μm^2^ Each

**Fig. 2:**
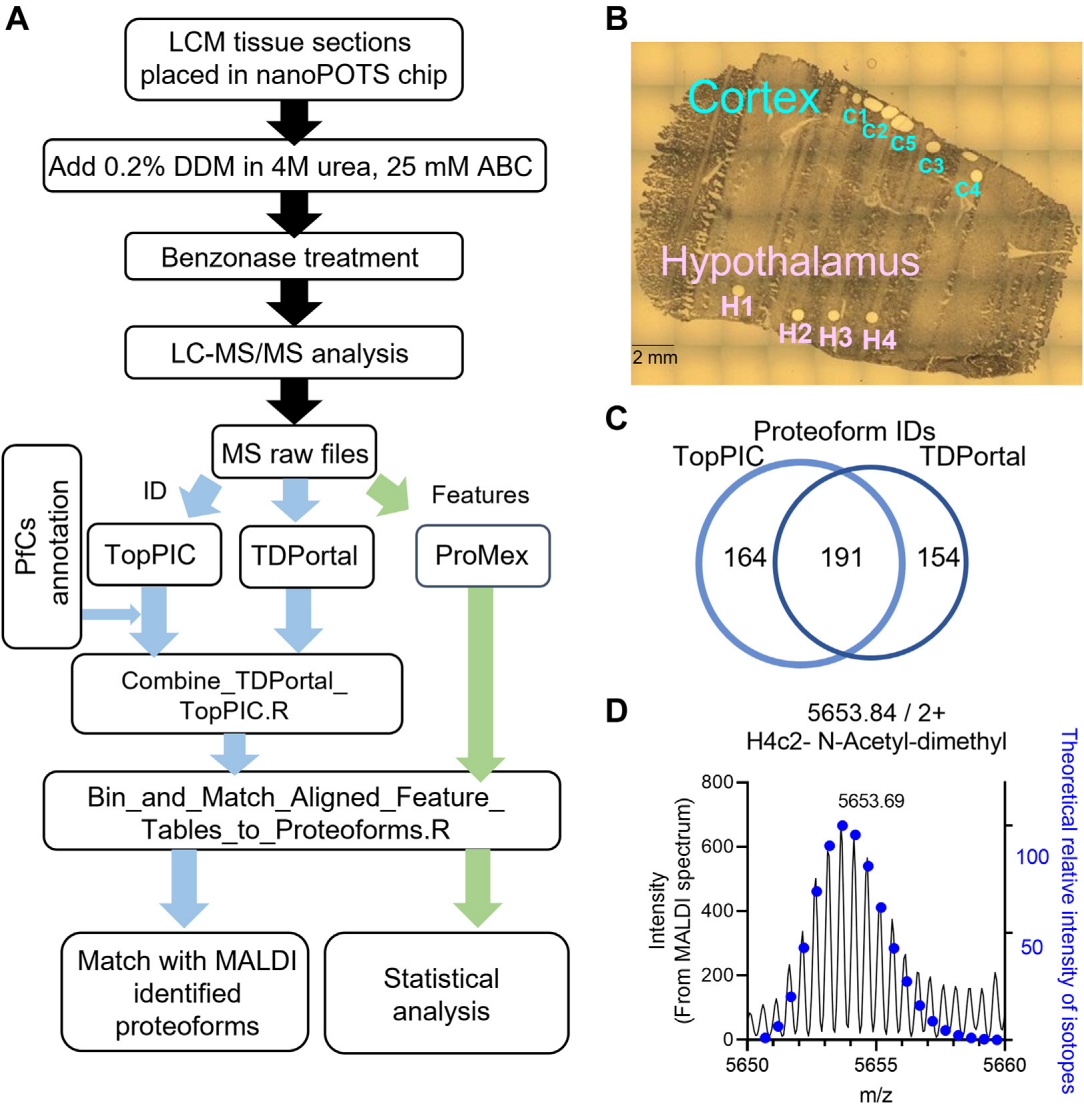
**Overall workflow for quantitative top-down proteomics analysis of rat brain tissue sections.***A*, workflow of processing LCM-derived tissue samples with nanoPOTS-TDP platform. *B*, optical image of rat brain tissue section showing where the small LCM punches were taken in the cortex and hypothalamus regions. *C*, Venn diagram showing the overlap of quantifiable proteoforms across all samples by TopPIC and TDPortal. *D*, Zoom-in view of the MALDI intact protein spectrum for the histone H4 proteoform, which was assigned based on identification by nanoPOTS LC-MS/MS. ABC, ammonium bicarbonate; DDM, n-dodecyl-beta-maltoside; LCM, laser capture microdissection; nanoPOTS, nanodroplet processing in one-pot for trace samples; PfCs, proteoform clusters; TDP, top-down proteomics; TopPIC, top-down mass spectrometry–based proteoform identification and characterization.

We applied the improved nanoPOTS TDP protocol to study LCM-derived rat brain tissues from cortex and hypothalamus regions. In these analyses, we employed FAIMS, which has been previously shown to improve proteoform coverage from bulk brain tissues (30). The top-down workflow, illustrated in Figure 2*A*, involved proteoform identification using two software tools (TopPIC (34) and TDPortal (38)); proteoform clustering to minimize redundancy using TopPICR; proteoform quantitation with ProMex; and data integration using custom R scripts.

We sectioned and separately analyzed five spots in the cortex and four spots in the hypothalamus with an area of ∼100,000 μm^2^ each (Fig. 2*B*), corresponding to roughly 200 cells (a mixture of neurons and immune cells). In the raw data, we observed a cluster of peaks with high intensities near 6.5 kDa in all analyses, which were not identified by the database search. With manual analysis of fragmentation data, we assigned these signatures to aprotinin, one of the ingredients from protease inhibitor cocktails we added in the lysis buffer. While these species did not directly interfere with the analysis, their high abundance suppressed endogenous proteoform signals and reduced MS/MS time available for their characterization, outweighing the benefit of protease addition. This finding corroborates a recent TDP study (30), which mentioned some protease inhibitor cocktails branded as MS-compatible contain small proteins and should be carefully considered for TDP applications.

The initial output from TopPIC and TDPortal listed 621 and 925 proteoforms, respectively. The two search engines have complementary algorithms but also feature different scoring and formatting, making it difficult to directly compare the results. To leverage complementarity and enhance proteoform coverage, we combined identifications from TDPortal and TopPIC that passed 1% FDR as defined by each tool. In parallel, ProMex was used to quantify proteoform features at the MS1 level independent of the identifications from the MS/MS data. Detected features were also aligned across all the samples using ProMexAlign algorithm. This alignment step, which is similar to the commonly used match-between-run (52, 53) or accurate mass and time tag (54) approach in BUP, was particularly important for filling the missing values in quantitative analysis. The aligned feature abundances were then attached to the combined proteoform identifications based on accurate mass and RT matching. With this data integration approach, we obtained 509 quantifiable proteoforms (supplemental Fig. S2). These included 191 proteoforms identified by both TopPIC and TDPortal, 164 identified only by TopPIC, and 154 identified only by TDPortal (Fig. 2*C*). Our workflow relied on the generic data of accurate mass, RT, and identification, and it thus can be applied to other TDP software outputs (such as identifications by pTop (55) or ProSightPC (56) and feature abundances from FLASHDeconv (57)).

Combining the identifications from these two complementary tools resulted in a higher number of total proteoform counts, but caution must be taken when merging the results. The major challenge is the split of proteoform abundance into multiple isotopologs for the same proteoform due to deisotoping error in the deconvolution step. To minimize redundancy, we chose to cluster LC-MS features within 15 ppm mass tolerance while considering deisotoping error and ± 4 min RT to best accommodate the results from TopPIC and TDPortal with different distributions. The rationale for the selection of these parameters was described with more details in supplemental Fig. S3. A balance was needed to minimize redundant proteoforms, while not over-merging unique proteoforms with small differences in mass and RT. Open modification search tool such as TopPIC can be particularly susceptible to redundant proteoforms, because deisotoping error could be assigned as a unique proteoform with unexpected mass shifts. Using a large mass error tolerance window of ± 1 Da can minimize the redundancy from deisotoping error, but with added risk of merging unique proteoforms with small mass differences (supplemental Fig. S3*A*). Within TopPIC, an “adjusted mass” was reported in addition to the experimental “precursor mass”. This adjustment reduced the deisotoping error for proteoforms without unexpected mass shifts but also introduced variations in the reported mass (supplemental Fig. S3*B*). We tested the use of either adjusted mass or experimental precursor mass from TopPIC using otherwise identical parameters for merging redundant features. Our manual analysis revealed using the “adjusted mass” showed fewer redundant features than using the “precursor mass” (supplemental supplemental Fig. S3*C*). The two approaches showed decent overlap of matched features by intact mass (supplemental Fig. S3*D*). Most unique features were due to deisotoping error and eventually matched to the same proteoforms (supplemental Fig. S3*E*), with only minor changes to the abundance values (supplemental Fig. S3*F*). Considering the narrow mass error tolerance of 15 ppm used in our filtering, we selected the “precursor mass” for comparing with masses reported by ProMex in the following discussion. The disadvantage was the additional redundant proteoforms that need to be manually merged primarily due to deisotoping error and occasionally also due to discrepancy in the proteoform identifications. Improved deisotoping algorithms (33, 57) and more robust proteoform FDR definitions (58) are needed to more effectively handle the ambiguity that is often seen for low abundance MS1 features and low quality MS2 data. Using the defined parameters, the final list of quantified proteoforms were mostly showing mass error <5 ppm (supplemental Fig. S3*G*), and RT <2 min (supplemental Fig. S3*H*).

The region-specific LC-MS/MS data can be used to generate spatially resolved proteoform databases for assigning peaks in MALDI-MSI data (24), where MS/MS data are typically limited or absent. Figure 2*D* shows an example of the highly abundant doubly charged peaks near *m*/*z* 5653.81 in an averaged MALDI spectrum from rat brain, which can be assigned as H4c2[N-acetyl&dimethyl] (5650.69 monoisotopic, *charge 2+*) using the LCM-nanoPOTS-TDP data from similar rat brain sections (Fig. 2*D* blue dots). Encouragingly, all major peaks in the full MALDI spectrum could be annotated with proteoform identifications from nanoPOTS data (supplemental Fig. S4). In MALDI-MSI applications, the singly charged or doubly charged protein ions can be recalcitrant to fragmentation. Hence, proteoform assignments in MALDI-MSI often rely on global TDP data generated using bulk samples or complementary data from *in situ* digested peptides (59, 60). Recent human proteoform atlas building efforts have been fruitful in generating tissue and cell type–specific proteoform databases (61, 62, 63), but they may not fully represent the proteoform subpopulations in specific tissue regions. The proteoform profile may change in different microenvironments, and these differences can remain hidden in bulk analyses due to “signal dilution”, where bulk analyses average the response of entire tissues, obscuring region, and cell-specific responses. Therefore, a spatially resolved proteoform database from nanoPOTS (or microPOTS) TDP could be highly valuable for accurate assignment of proteoforms in different tissue functional units and cells. Our future work will investigate the quantitative correlation between MALDI-MSI and TDP data from matching LCM regions.

### LCM-NanoPOTS-TDP Captured PTM and Isoform Information

**Fig. 3:**
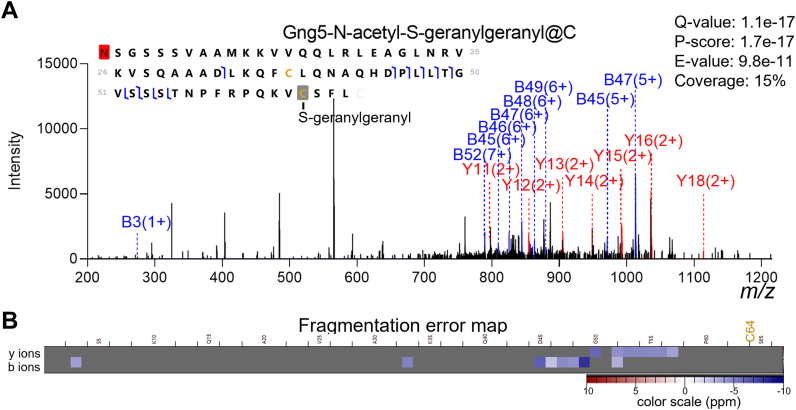
**A representative modified proteoform of Gng5.** (*A*) Tandem mass spectrum with annotated fragments and (*B*) fragment error map. Despite the relatively low sequence coverage, the b/y ions supported assignment of N-terminal acetylation and S-geranylgeranyl modification at the cysteine near the C-terminus (scan #3185 in Hubmap_Intact_Brain_C1_CV40.raw). The unlabeled peaks < m/z 600 were presumably from other co-isolated species (supplemental Fig. S5).

The majority (∼70%) of our identified proteoforms were unmodified (not counting backbone cleavages and N-terminal acetylation), concurring with ∼24% modified proteoforms in a recent TDP study of bulk human tissues (64). Nonetheless, several interesting modified proteoforms were confidently identified. For example, we identified Gng5 (guanidine nucleotide-binding protein G(I)/G(S)/G(O) subunit gamma) with S-geranylgeranyl modification at C64 (Fig. 3*A*), in agreement with previous reports (65) and the UniProt protein database. The unassigned fragments with high intensity at *m/z* 400 to 600 had mass differences matching to hexoses. They were likely originated from co-isolated species and cannot be easily explained by the assigned proteoform (supplemental Fig. S5). The unique benefit of TDP is the straightforward identification of proteoforms that can be challenging to differentiate using peptide-only data. In our results, myelin isoform 4 (P02688–4) was the only proteoform confidently assigned among the five recorded isoforms in UniProt. The other isoforms are results of alternative splicing and are only missing segments of the canonical sequence. Several myelin isoform 4 proteoforms with known PTMs were also detected with high confidence (proteoform level 1 or 2A). Distinct spatial distribution of myelin isoforms has been reported by nanospray desorption electrospray ionization measurements (66, 67). We found that Mbp-o-phospho has higher abundance in the cortex than in the hypothalamus, which is consistent with a previous study (66). These findings demonstrate TDP could play important role in deciphering proteoform-specific information, which is critical for understanding the contributions of proteoforms to cellular heterogeneity and function.

### LCM-NanoPOTS-TDP Captured Differential Proteoform Profiles in the Cortex and Hypothalamus Regions of Rat Brain

**Fig. 4:**
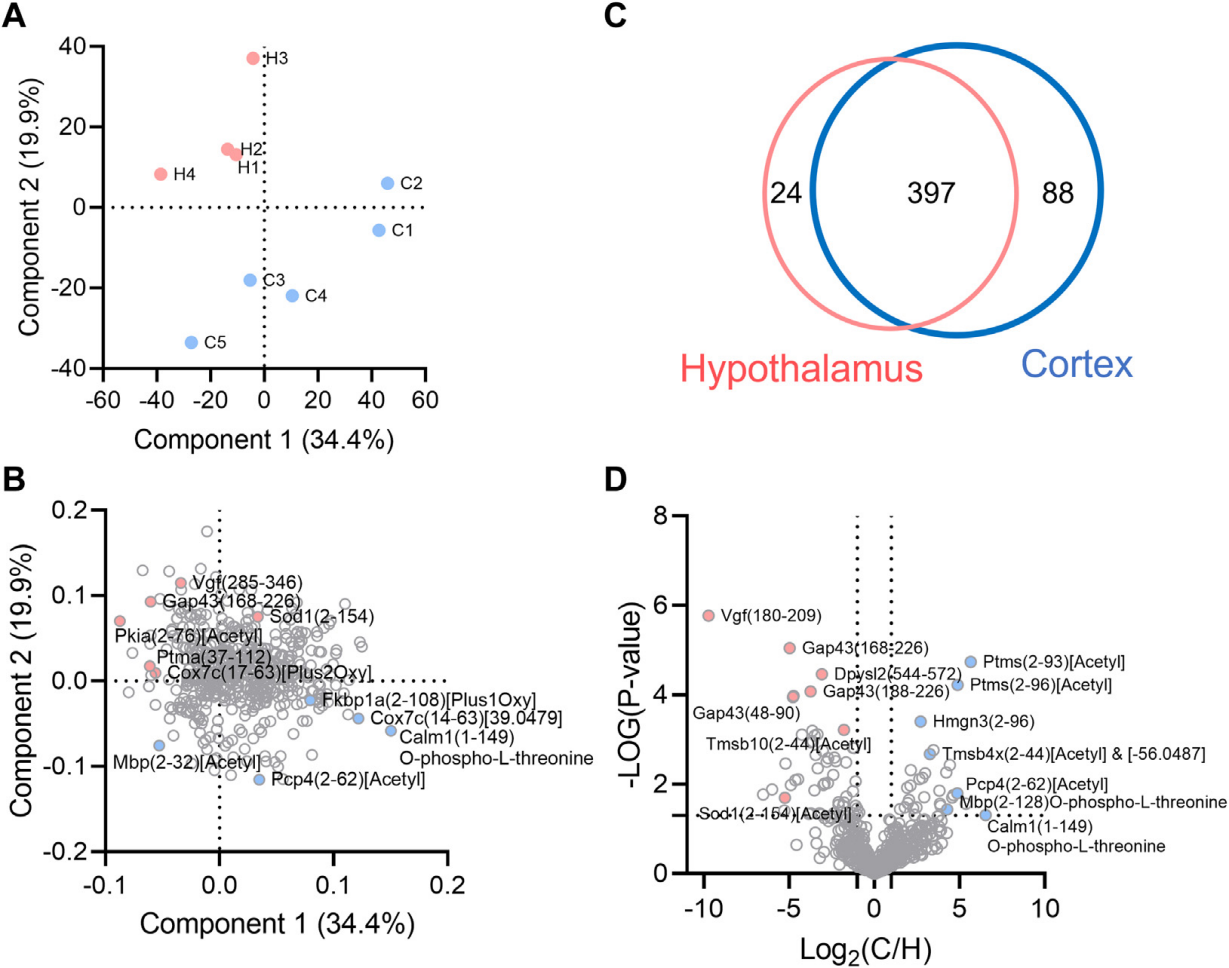
**Statistical analysis of protoeform abundances between cortex and hypothalamus.** Principal component analysis (PCA) of proteoform abundances yields (*A*) two distinct clusters of cortex (*blue*) and hypothalamus (*pink*) samples, and (*B*) candidate proteoforms for differentiating brain tissue types. *C*, identified proteoform numbers in cortex (*blue*) and hypothalamus (*pink*). *D*, volcano plot for proteoform in cortex and hypothalamus. Proteoforms are named as gene name, followed by starting and ending residue numbers in *parentheses*, and PTM (if any). PTM, posttranslational modification.

LCM-nanoPOTS-TDP captured different proteoform compositions in the cortex and hypothalamus regions based on the PCA where samples from the cortex and hypothalamus were grouped in blue and pink clusters, respectively (Fig. 4*A*). Variances in the nearby spots of the same tissue region implied potential heterogeneity even within the same region. The score plot of PCA (Fig. 4*B*) showed the differentiating proteoforms for the two tissue regions. Calm2-(1–149)O-phospho, Snca(1–140)[Acetyl], Pcp4(2–62)[Acetyl], and Mbp(2–128)O-phospho were enriched with cortex region, while Sncb(84–134), Vgf(285–346), Gap43(188–226), and Gap43(48–90) were enriched with hypothalamus regions. PCA analysis without data imputation showed the same trends (supplemental Fig. S6).

**Fig. 5:**
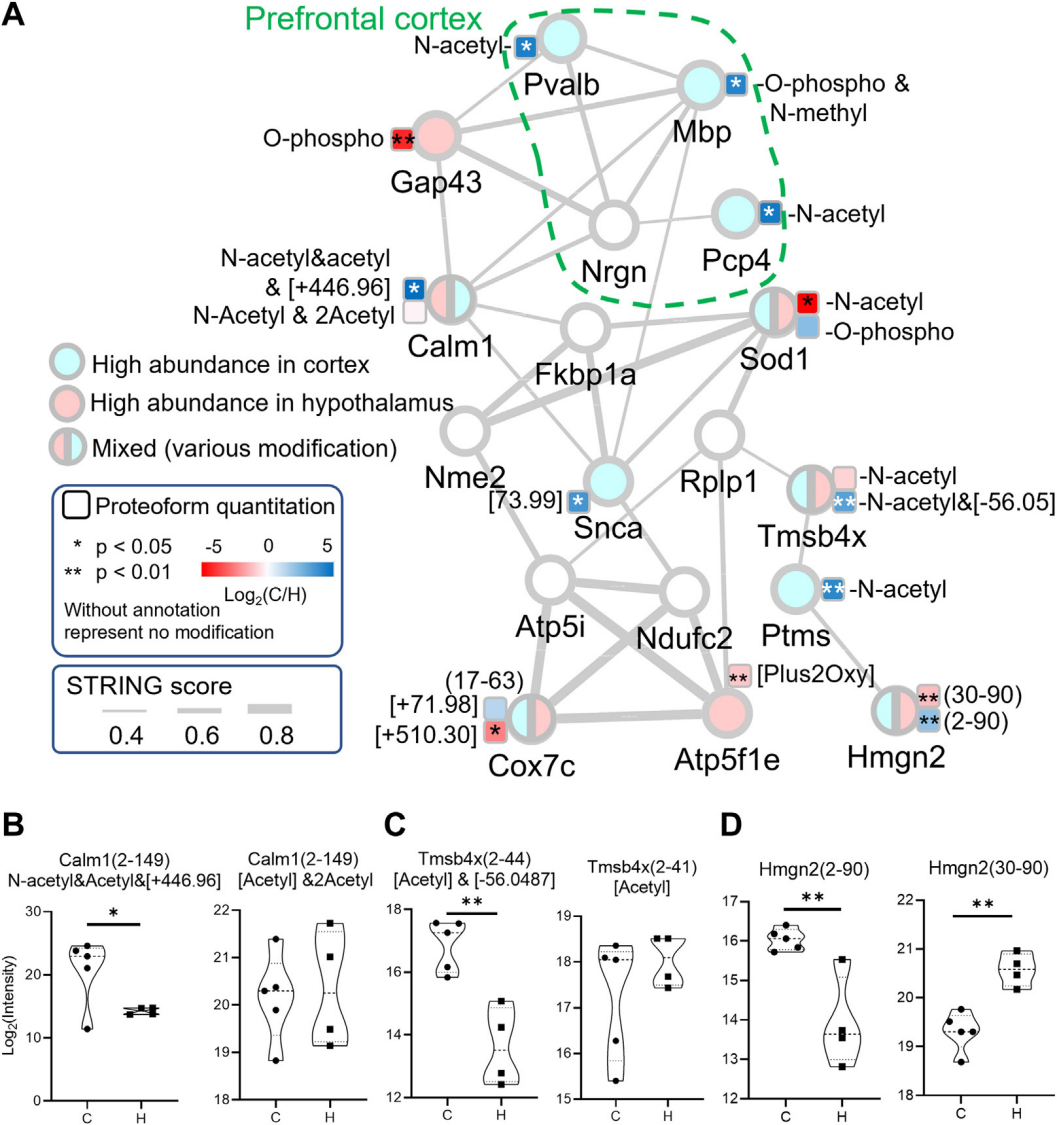
**Quantitative analysis of representative proteoforms inthebrain mapped to protein-protein interaction network.***A*, several proteoform clusters revealed significant differences in the protein–protein interaction network between the cortex and hypothalamus region. Proteins either had higher abundance in the cortex (*light blue*), hypothalamus (*pink*), or had mixed abundance profiles between the two regions. The *box* next to the *circle* corresponds to one representative proteoform for the protein with lowest *p*-value, which is colored with log2(C/H) with *dark blue* for higher expression in the cortex and *red* with higher abundance in the hypothalamus. In the case of proteins with mixed abundance profiles, two proteoforms with the lowest *p*-value and enriched in the cortex and hypothalamus were shown. Each *line* between proteins has interaction evidence in the String database. *B*, violin plots showing the abundances of Calm2-N-acetyl& 2 acetyl and Calm2-N-acetyl&acetyl&[+446.956], (*C*) Tmsb4x N-acetyl and Tmsb4x N-acetyl & [−56.05], as well as (*D*) Hmgn2(2–90) and Hmgn2(30–90) in the cortex: C and hypothalamus: H regions.

To investigate possible connections between PTMs, proteoforms, and spatial abundance differences, we mapped the proteoforms to the PPI database with the network plot in STRING (Fig. 5). Because some of the truncated proteoforms may be result of sample degradation, we further filtered the identified proteoforms to include only proteoforms covering over 60% of the canonical sequence from Uniprot protein database. In addition, only proteoforms from genes categorized as highly expressed in the brain were included. We selected one proteoform with the lowest *p*-value (*i.e*., most significantly changed in abundance between the two tissue regions) to represent each protein (Fig. 5). Several proteins (*e.g.*, Pvalb, Mbp) were known to be highly expressed in the prefrontal cortex (highlighted by green dash lines) in the tissue expression database(TISSUES) (45). We observed significantly higher abundances of their proteoforms in the cortex (blue circles in Fig. 5*A*), validating that our method captured the expected proteome differences between the two tissue regions.

While many identified proteoforms derived from the same gene had similar abundance profiles, some proteoforms showed opposite patterns (*e.g*., circle filled with half red and blue in Fig. 5*A*), implying different proteoforms could have distinct functions in different tissue regions. For these genes, we selected two representative proteoforms with the lowest *p* value in each direction of the abundance profile change (*i.e.*, blue indicates enrichment in cortex, and red indicates enrichment in hypothalamus). For example, two most significantly differentiating calmodulin proteoforms (Fig. 5*B*) showed different abundance profiles, with Calm1[N-acetyl&acetyl&446.96] being highly abundant in cortex (*p* = 0.0175) and Calm1[N-acetyl&2acetyl] being highly abundant in hypothalamus (*p* = 0.194). Calm1 is known to interact with both Gap43 and Mbp (myelin basic protein), whose major proteoforms also showed opposite abundance profiles. Mbp [N-methyl&O-phospho] showed significantly higher abundance in cortex (*p* = 0.0044), suggesting a positive correlation with Calm1[N-acetyl&acetyl&446.96]. In contrast, Gap43[O-phospho] showed higher abundance in the hypothalamus (*p* = 0.0055). Both Calm1 and Gap43 are involved in filopodia growth in neurons (68). Phosphorylation of Ser41 on Gap43 eliminates calmodulin binding (69) and stabilizes the interaction of Gap43 with actin filaments (68), leading to increased membrane tension and promotion of filopodia growth (70). Therefore, the higher abundance of Gap43[O-phospho] may be related to the enhanced filopodia in hypothalamus relative to cortex. Moreover, calmodulin is a Ca^2+^ sensor, which means if its calcium binding pocket is blocked, the binding affinity of Ca^2+^ will reduce. The released calcium could stimulate phosphorylation on myelin protein (71) by calcium/calmodulin-dependent protein kinase (72). The lack of confident PTM assignment for Calm1[N-acetyl&acetyl&446.96] (supplemental Fig. S7) prevented us from interpreting the data under biological context. Yet, the spatially different abundance of Calm1[N-acetyl&acetyl&[+446.956] and Calm1[N-acetyl&2acetyl] suggested the proteoforms derived from the same gene (protein) have different functional roles in the cortex and hypothalamus regions.

Several other proteoforms and their interacting partners also had unknown PTMs (*i.e.*, not assignable within the scope of this study). They were simply annotated as mass shifts here (see representative spectrum for Tmsb4x in supplemental Fig. S8). Some of the unknown shifts may originate from noncovalent adducts or labile PTM (which was lost during fragmentation, *e.g.*, supplemental Fig. S9 describing Cox7c proteoforms), with their biological significance currently unknown. The ambiguities in PTM assignment and localization largely originated from insufficient sequence coverage in MS2 spectra, which can be improved by employing alternative fragmentation methods, such as electron transfer dissociation or ultraviolet photodissociation. A larger number of datasets is also needed to better define the statistical significance. For example, the Tmsb4x (2, 3, 4, 5, 6, 7, 8, 9, 10, 11, 12, 13, 14, 15, 16, 17, 18, 19, 20, 21, 22, 23, 24, 25, 26, 27, 28, 29, 30, 31, 32, 33, 34, 35, 36, 37, 38, 39, 40, 41) Acetyl&[-56.05] proteoform showed significant difference in abundance between the two tissue regions, while the Tmsb4x (2, 3, 4, 5, 6, 7, 8, 9, 10, 11, 12, 13, 14, 15, 16, 17, 18, 19, 20, 21, 22, 23, 24, 25, 26, 27, 28, 29, 30, 31, 32, 33, 34, 35, 36, 37, 38, 39, 40, 41) Acetyl proteoform showed a large variation in abundance within the cortex group and no significant difference with the hypothalamus group (Fig. 5*C*). While experimental variation can simply explain the lack of statistical significance, microheterogeneity within the same tissue region may also play a role and could be investigated in future studies.

Another noteworthy pair of proteoforms with distinct abundant profile was the full-length and truncated Hmgn2 (MS2 spectra in supplemental Fig. S10). Hmgn2(2–90) had higher abundance in the cortex, and N-terminally truncated Hmgn2(30–90) was higher in hypothalamus (Fig. 5*D*). Hmgn2 has been reported to have high abundance in the cortex in human protein atlas database (73). Hmgn2(30–90) lacking part of nucleosome binding domain could have altered activity related to regulation of chromatin structure, transcription, and DNA repair (74). The truncation could have been regulated *via* specific proteases. TDP readily captured such events and may help elucidate new mechanisms.

We compared our TDP data to a similar nanoPOTS-BUP study which had a total of 956 protein identifications (75). (supplemental Fig. S11) Only 53 proteins were identified in both experiments. The low overlap was not uncommon as was previously reported (76). Additionally, BUP and TDP data were derived from different regions of the brain tissue in two independent studies. TDP covered ∼20% of BUP identified proteins, with major gap in capturing bigger proteins. Combined use of multiple protease digests would be needed to confirm the PTMs identified in TDP when integrating TDP and BUP data. Among the overlapping proteins, TDP offered high coverage to define the starting/ending residues of proteoforms, whereas most BUP identifications had peptides covering <50% of the protein sequence. For the 162 uniquely identified proteins in TDP, ∼50% were full length proteoforms and not simply degradation products, suggesting TDP is more sensitive in capturing small proteins and their proteoforms than BUP. Nonetheless, our current study demonstrated the potential of integrated LCM-nanoPOTS-TDP and MALDI-MSI platforms for quantifying proteoforms in a spatially resolved manner. The distinct abundance profiles for several proteoforms originating from the same gene reinforce the importance of proteoform-specific measurements to precisely define their functional roles.

## Discussion

In this study, we improved our previous nanoPOTS-TDP protocol for small sample analysis and applied it to quantitative TDP study of LCM-derived rat brain tissue sections. The use of benzonase in the extraction step improved proteoform counts by efficiently digesting DNA polymers and releasing DNA-binding proteins. We also streamlined the data analysis workflow by integrating several TDP software tools. The R scripts (37) combined and clustered proteoform identifications from TopPIC (34) and TDPortal (38) outputs to maximize proteoform coverage and minimize redundancy. Independently, proteoforms were quantified at the MS1 level using ProMex (39) and aligned across all datasets to reduce missing values. The proteoform identifications were then combined with their corresponding abundances for label-free quantitation. Our data analysis workflow is generic and can be readily adapted to other TDP software outputs. Overall, we obtained 509 quantifiable proteoforms across cortex and hypothalamus regions of rat brain. The abundance profiles facilitated elucidation of proteoforms’ function connecting with PPI network databases. Notably, we observed different abundance profiles among several proteoforms derived from the same gene, highlighting the need for the proteoform-aware mapping of tissues. Our future work will involve integration of LCM-TDP with MALDI-MSI for enhanced throughput and spatial resolution for proteoform imaging from tissues. We envision that spatially resolved TDP will become an essential tool for generating high confidence identifications and quantitation necessary for biomarker discovery, *e.g.*, higher throughput MSI experiments for precision diagnosis.

## Data Availability

The MS proteomics data have been deposited to MassIVE with accession MSV000089163. It includes MS raw data files, TDPortal search results of rat brain (supplemental Table 2), and TopPIC search results of rat brain (supplemental Table 3) and benzonase experiment (supplemental Table 4). The annotated MS/MS spectra from TDPortal results (open with TDViewer 2.0). and TopPIC results (“_html.zip” files) were also included.

## Supplemental data

This article contains supplemental data. (34, 37, 38, 39)

## Conflict of interest

The authors declare no competing interests.
