# Supplementary material for: Spatially Resolved Top-Down Proteomics of Tissue Sections Based on a Microfluidic Nanodroplet Sample Preparation Platform
Source: Mol Cell Proteomics. 2023 Jan 2;22(2):100491. doi: 10.1016/j.mcpro.2022.100491 (PMC9944986; doi:10.1016/j.mcpro.2022.100491)
Supplement: Supplemental Figures S1–S11 [file mmc1.docx]

Supporting Information

Spatially resolved top-down proteomics of tissue sections based on a microfluidic nanodroplet sample preparation platform

Yen-Chen Liao^1^, James M. Fulcher^1^, David J. Degnan^2^, Sarah M. Williams^1^, Lisa M. Bramer^2^, Dušan Veličković^1^, Kevin J. Zemaitis^1^, Marija Veličković^1^, Ryan L. Sontag^2^, Ronald J. Moore^2^, Ljiljana Paša-Tolić^1^, Ying Zhu^1,3*^, and Mowei Zhou^1,*^

1. Environmental Molecular Sciences Laboratory, Pacific Northwest National Laboratory, 3335 Innovation Boulevard, Richland, Washington 99354, United States.

2. Biological Sciences Division, Pacific Northwest National Laboratories, 902 Battelle Boulevard, Richland, Washington 99354, United States.

3. Present address: Department of Microchemistry, Lipidomics and Next Generation Sequencing, Genentech, 1 DNA Way, South San Francisco, 94080, United States.

^*^Correspondence: Dr. Mowei Zhou, [mowei.zhou@pnnl.gov](mailto:mowei.zhou@pnnl.gov)

Dr. Ying Zhu, [zhu.ying@gene.com](mailto:zhu.ying@gene.com)

Includes Figure S1-S11.


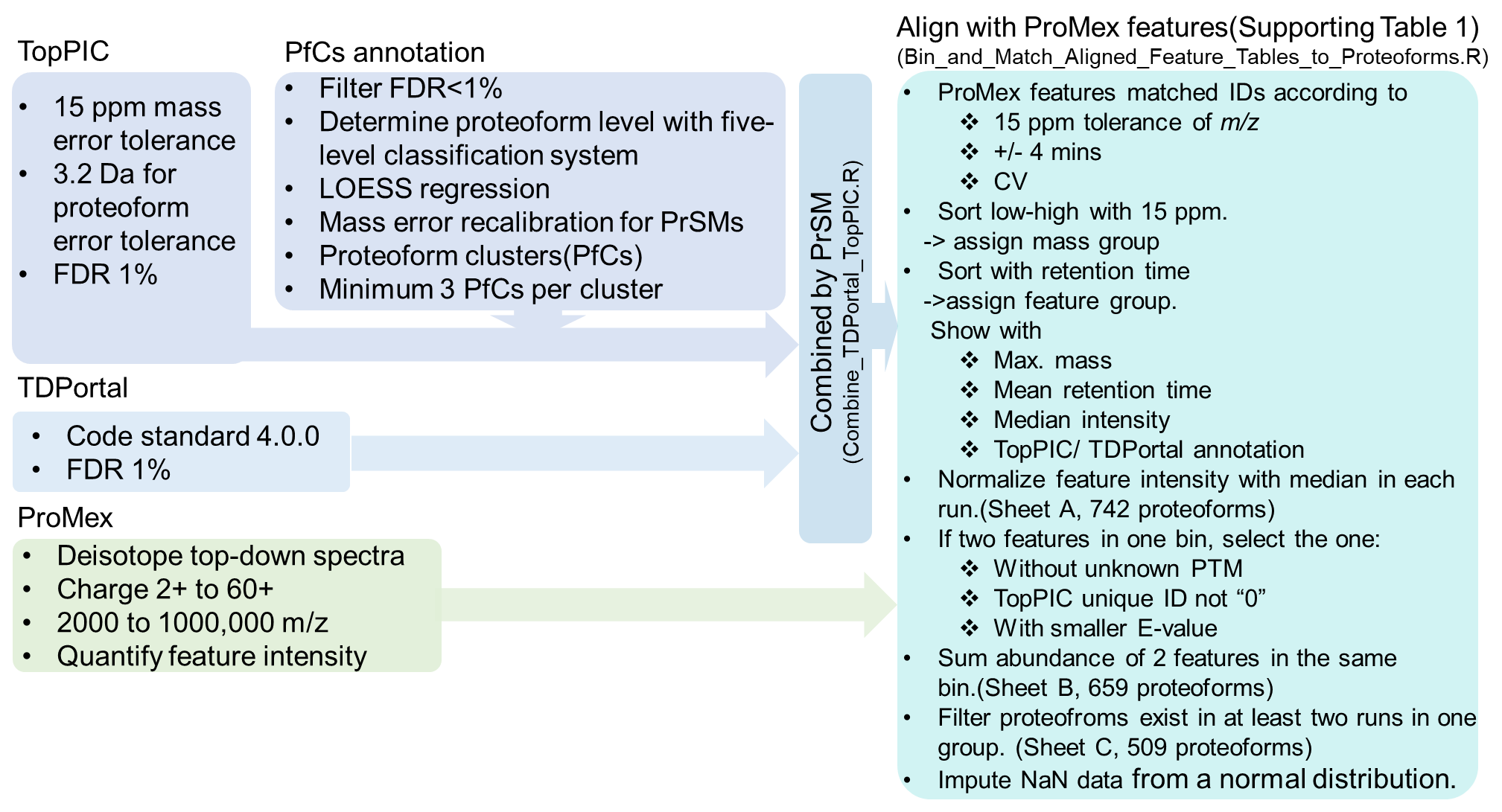


**Supporting Figure S1**. **Workflow for data processing with detailed parameters.** The programs are in the links below: TopPIC(34) (<https://www.toppic.org/software/toppic/index.html>), TDProtal(38) (<https://nrtdp.northwestern.edu/tdportal-request/>), ProMex(39)(<https://github.com/PNNL-Comp-Mass-Spec/Informed-Proteomics/tree/master/ProMex>), TopPICR(37) ([https://zenodo.org/record/5826349#.Yy8ft3bMKuc](https://zenodo.org/record/5826349#.Yy8ft3bMKuc)), and align with ProMex features (<https://github.com/PNNL-HubMAP-Proteoform-Suite/spatially-resolved-TDP/tree/main/ProMexAlign_Proteoforms>). The final proteoform list and intermediate lists during the processing steps were included in the Supporting Table 1. Manual curation further reduced the redundancy in the output from 742 to 509 proteoforms. Ambiguous PTM assignments were also corrected to unknown mass shifts.

**Supporting Figure S2**. **Proteoform counts from 100 cells on polypropylene and glass nanoPOTS chips.** Polypropylene (PP) chips delivered a higher number of proteoform identifications than glass chips from samples containing ~100 HEK cells (n=5 for each condition). The improvement is due to reduced absorptive losses on PP surface in comparison to glass surface.


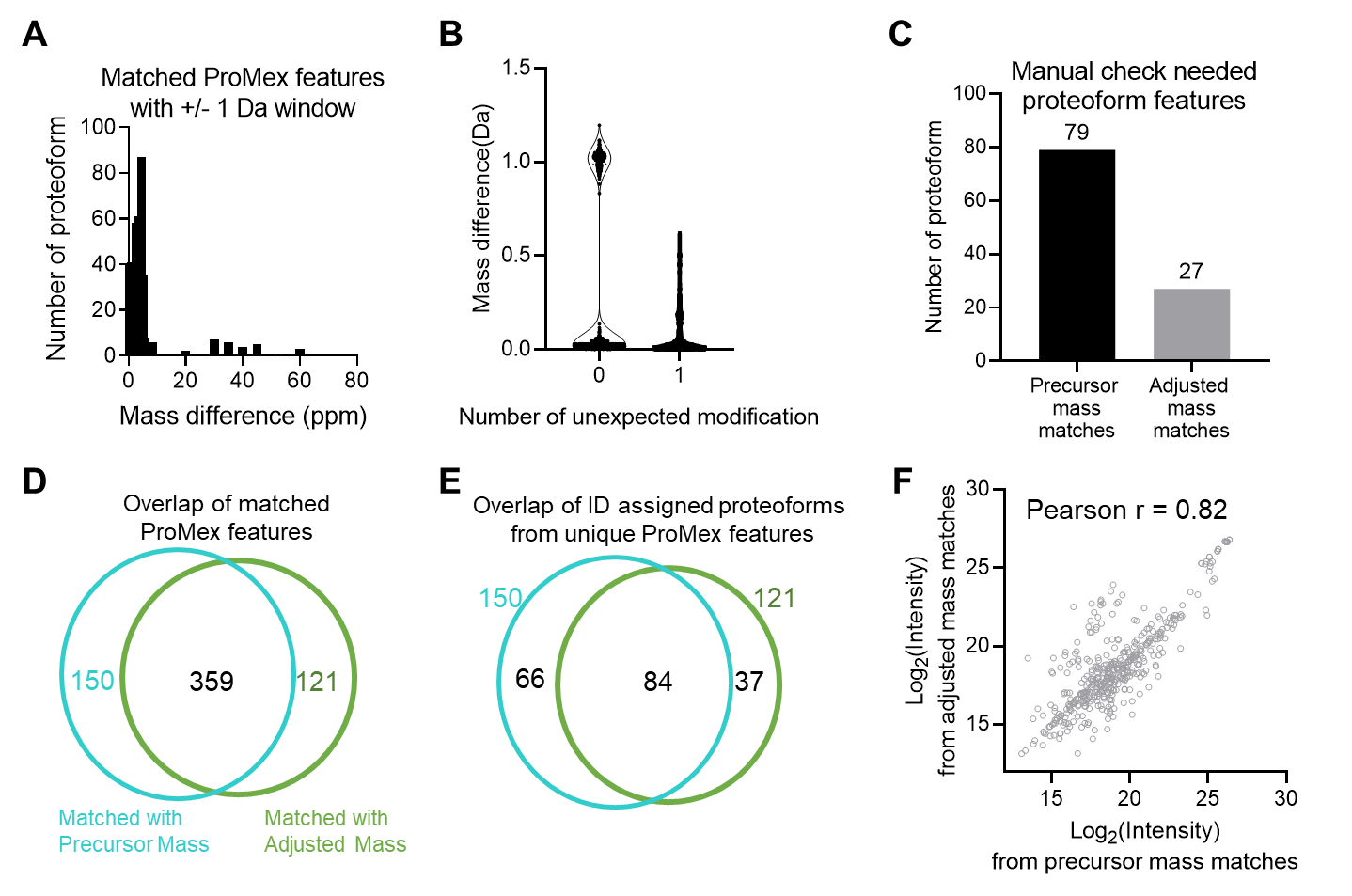


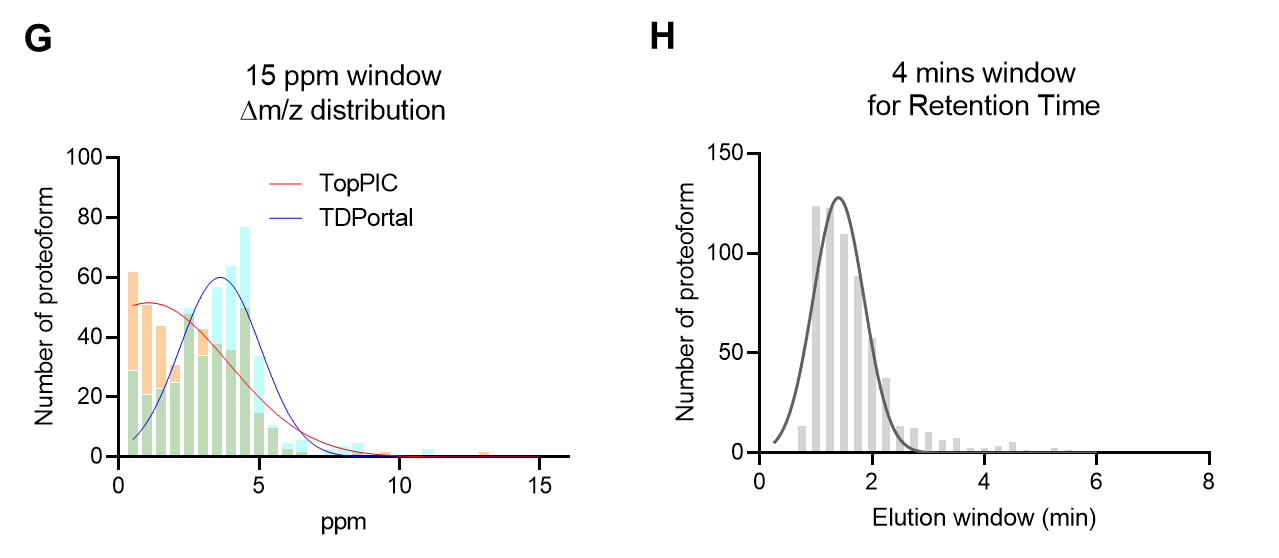


**Supporting Figure S3. Evaluation of parameters for merging redundant proteoform features.** *A*, When using a broad mass tolerance window of +/- 1 Da to minimize redundant isotopologues, a small distribution of ProMex features were matched with high mass error in 20-60 ppm range. *B*, TopPIC reports two different masses for each proteoform identification – “adjusted mass” and “precursor mass”. The absolute mass difference between these two are plotted for proteoform without and with unexpected mass shifts. The proteoforms without unexpected mass shifts showed a cluster near 1 Da mass shift, suggesting the “adjusted mass” attempted to correct some deisotoping error after considering the identified proteoforms. For proteoforms with unexpected mass shifts, there was no obvious cluster near 1 Da but instead a relatively broad distribution of mass differences. *C*, Manually checking the merged features identified additional redundant proteoforms. Using the adjusted mass yielded fewer redundant proteoforms from deisotoping error. *D,* Venn diagram showing the overlapping of matched ProMex features using the “precursor mass” and “adjusted mass” from TopPIC. *E,* Venn diagram showing the overlap of assigned proteoforms to the uniquely matched ProMex features, showing 84 common proteoform assignments. Many unique mass matches were simply from deisotoping error (i.e., differet isotopologs of the same proteoform). *F,* For the 84 shared proteoforms with different ProMex feature masses, the abundance values were linearly correlated, suggesting the isotopologs introduce relatively small change to the quantitative analysis. *G,* The mass error distribution of the assigned proteoforms in the final reported list from our workflow, most of which were < 5 ppm. *H,* The retention time window distribution (reported by ProMex) of the reported proteoforms, which are mostly < 2 min.


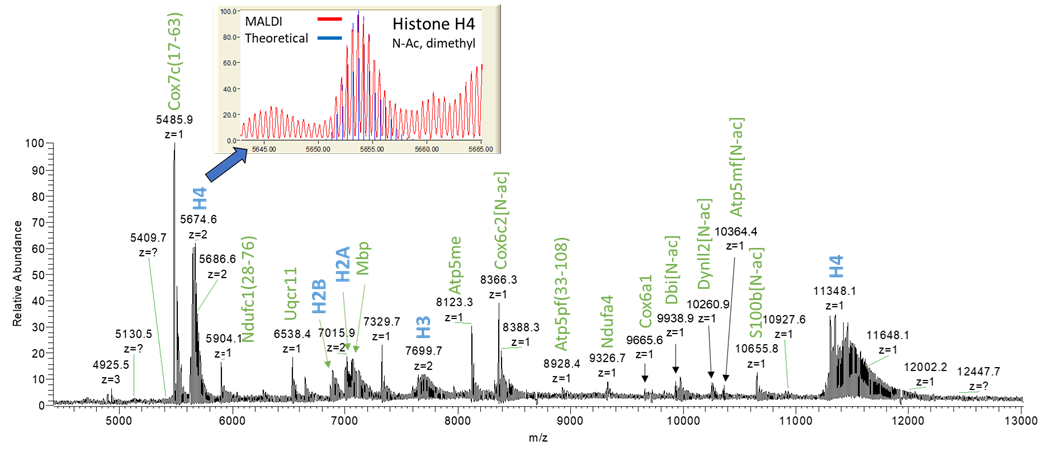


**Supporting Figure S4**. **Representative MALDI spectrum of intact proteins from rat brain section.** The data were acquired in MSI mode as described in the Experimetal section, and all pixels were summed to yield the spectrum shown. Major peaks were annotated with gene names (green text) based on the proteoforms identified in LCM- nanoPOTS. Truncated forms were labeled with the starting – ending residues in parentheses. PTMs were noted in brackets. Because multiple histones proteoforms corresponding to one or more histone genes were detected with similar masses, only the family names were labeled (blue text) for simplicity in this demonstration. The inset shows the zoom-in region of the MALDI spectrum (red trace) overlapping with the theoretical isotopic distribution of histone H4 N-ac, dimethyl proteoform (blue trace).


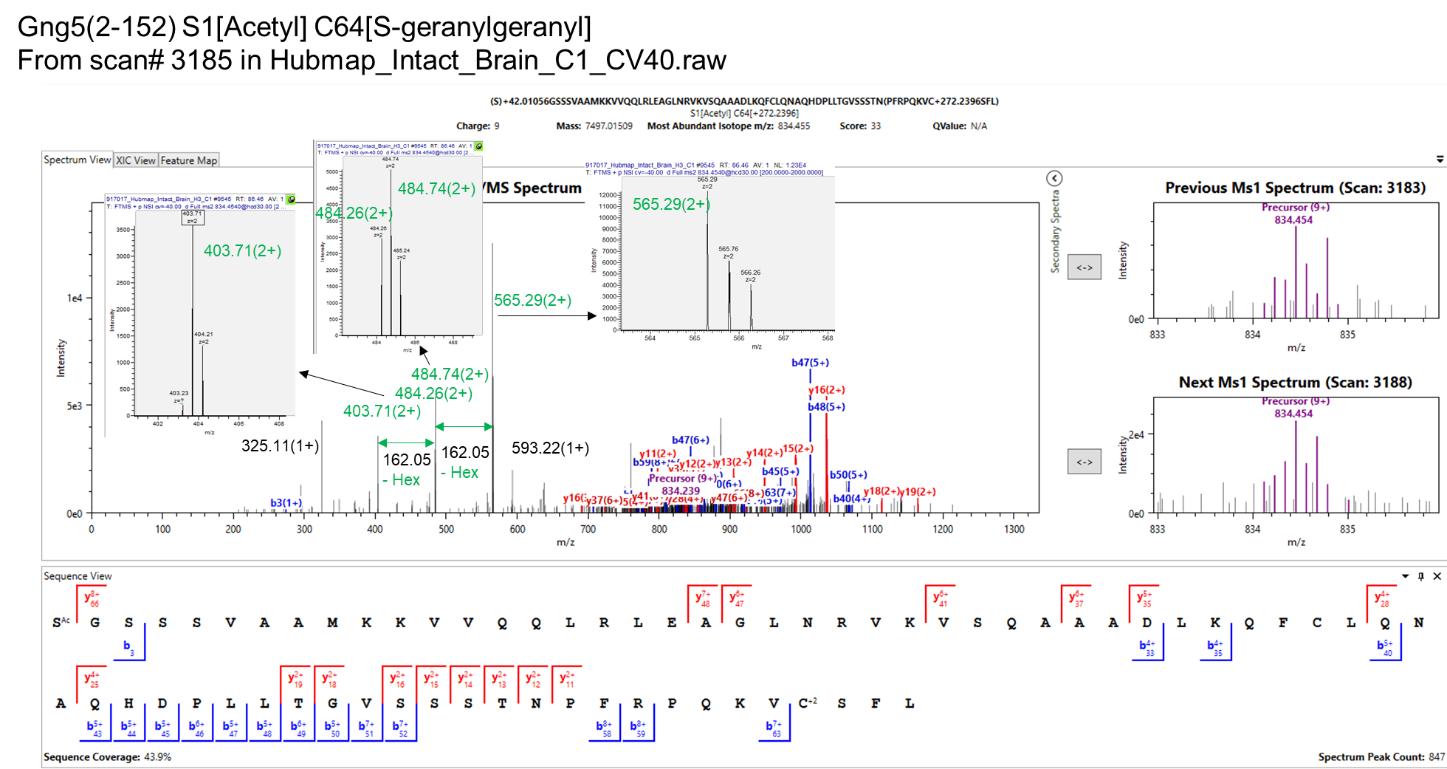


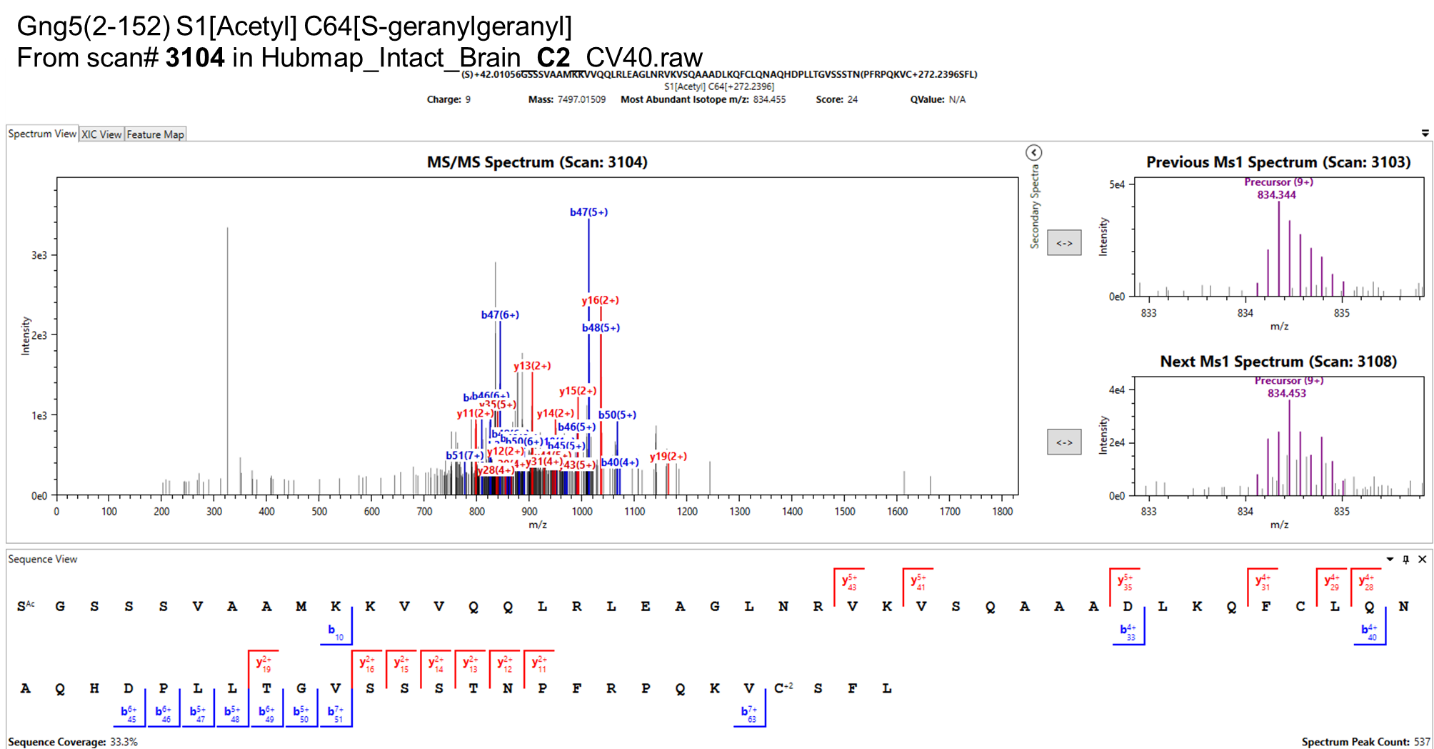


**Supporting Figure S5. Representative spectra of modified Gng5 in different runs.** In the MS2 spectrum of Gng5(2-152)S1[Acetyl]C64[S-geranylgeranyl], the un-matched base peak from scan#3185, including 565.29(2+), 484.74(2+), and 403.71(2+) could be fragments from co-eluting species. In another MS2 spectrum from C2_CV40.raw, we did not see the unassignable fragments at 565.29(2+), 484.74(2+), and 403.71(2+). The mass difference between these base peaks could be hexose(162.05 m/z), suggesting the co-eluting species may be related to glycans.

**
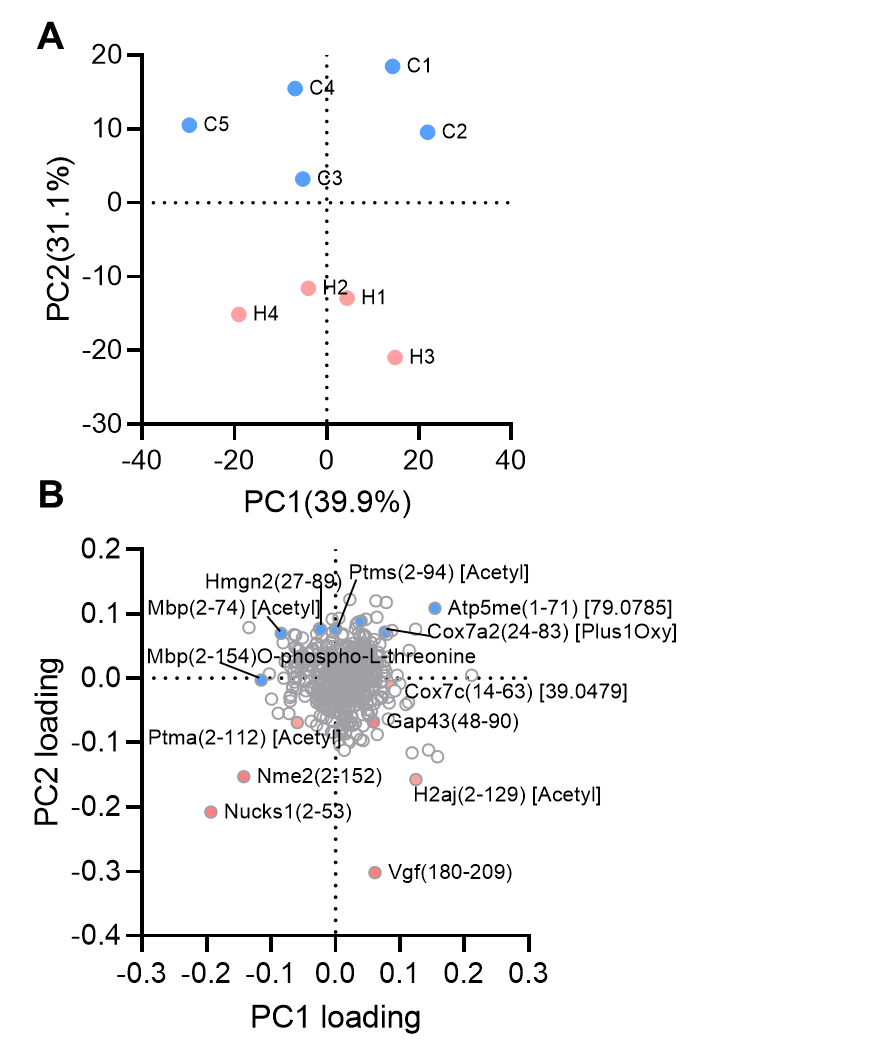
**

**Supporting Figure S6.** **PCA analysis without imputation.** *A,* PCA score plot. *B,* The loadings of PC1 and PC2. The loadings drove the separation to hypothalamus with Gap43(48-90), Vgf(180-209), Nme2(2-152), and Ptma(31-113); enriched in cortex with Mbp(2-74)[Acetyl], Hmgn2(27-89), and Atp5if1(27-107).The PCA separated the cortex and hypothalamus with PC2 as same as data-imputed PCA in Fig.3. Most protoefomrs in the loading plot , such as Gap43(48-90) and Vgf(180-209), have the same trend as the data-imputed PCA.


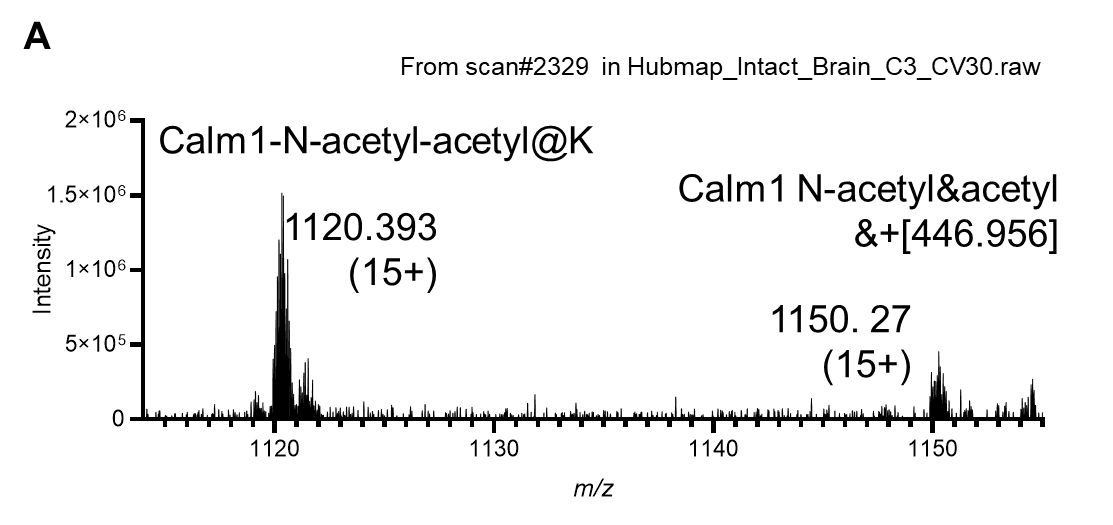


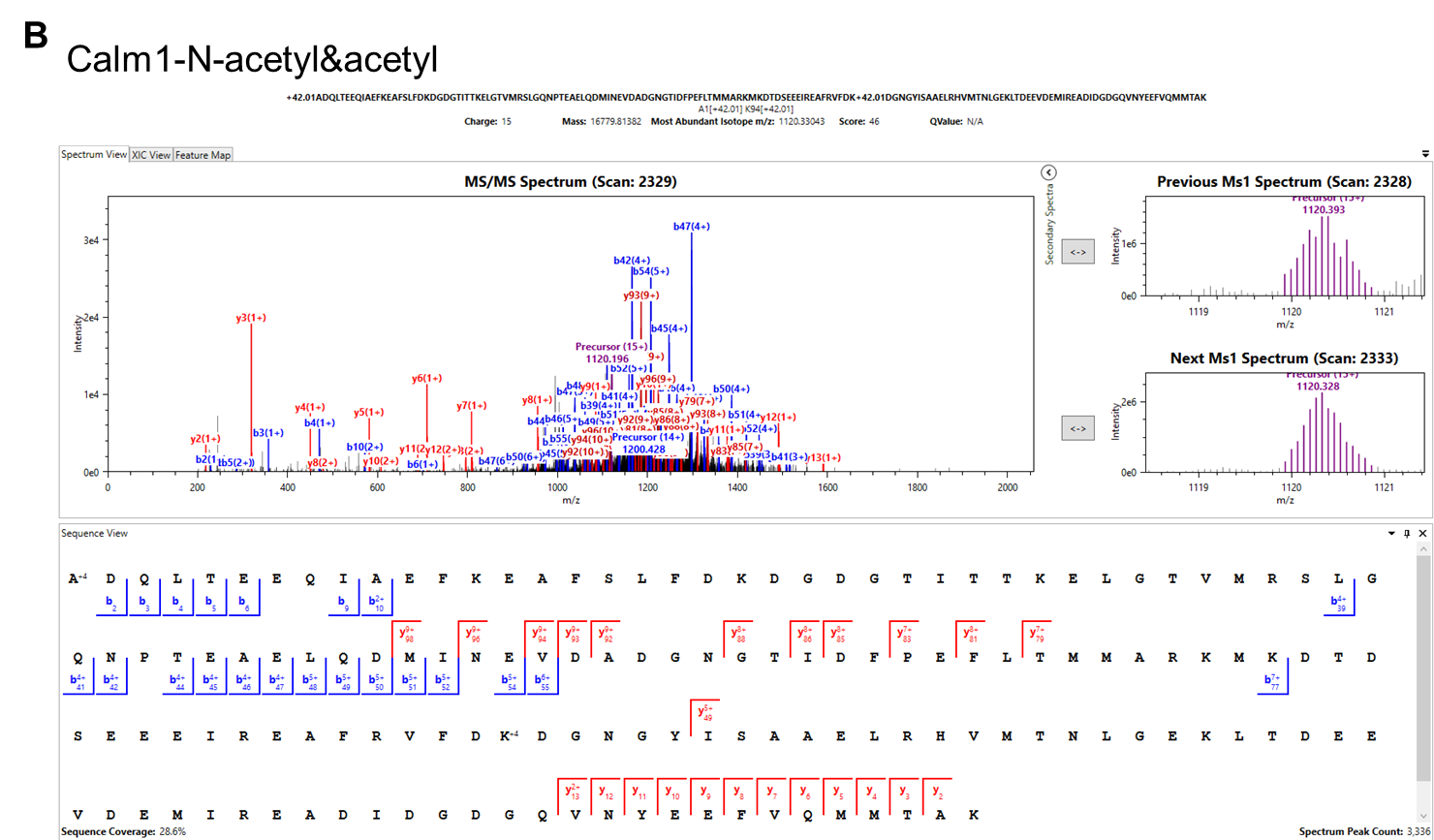


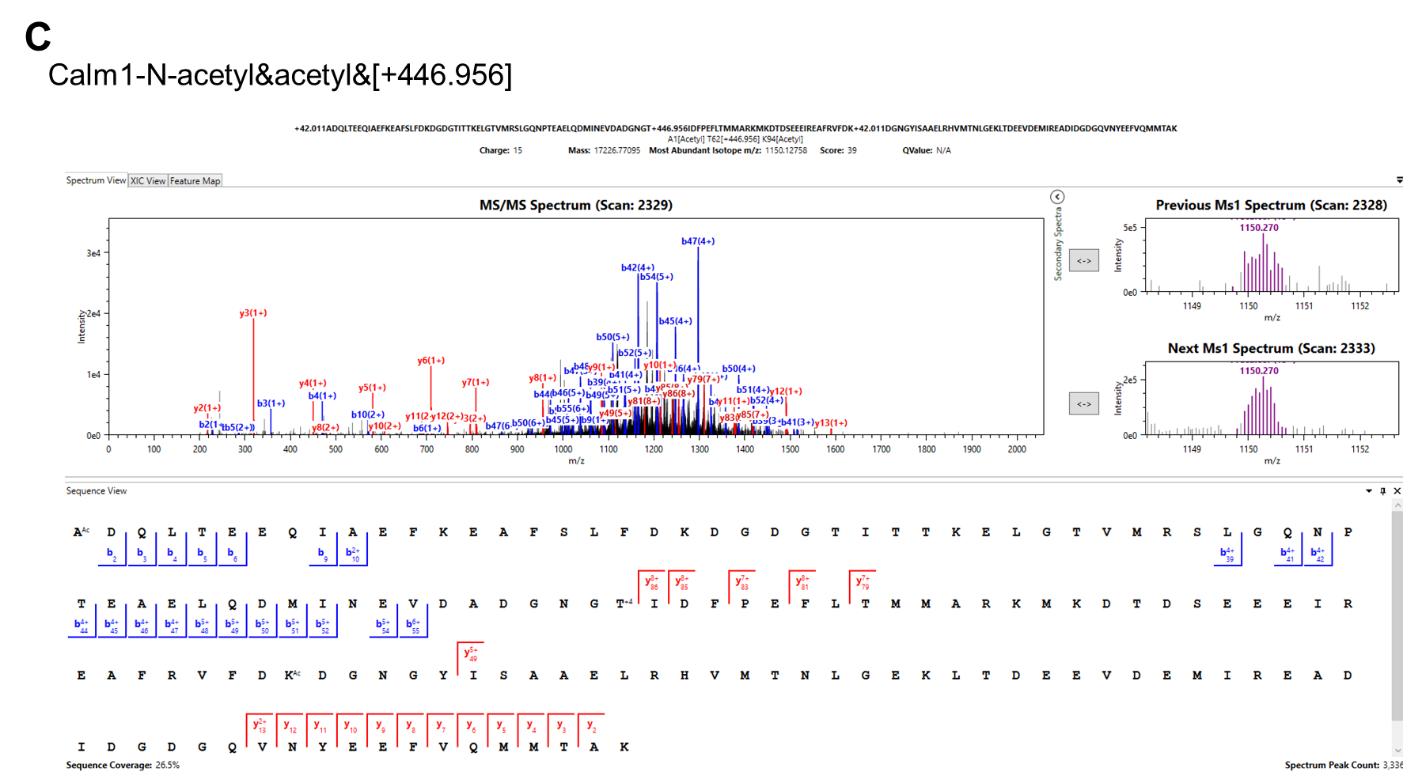


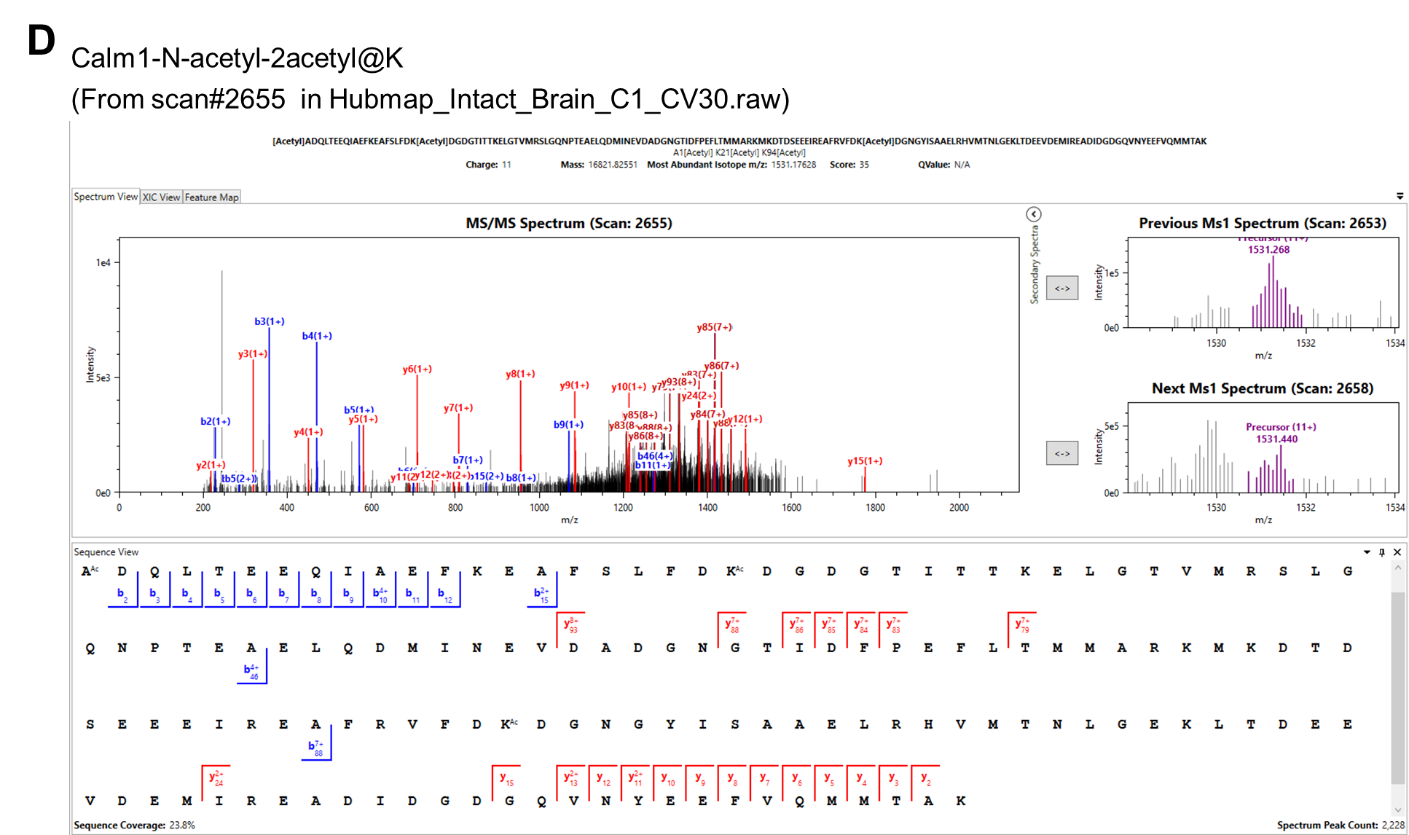


**Supporting Figure S7.** **Representative spectra from proteoforms of Calm1.** *A, MS* spectrum of Calm1-N-acetyl&acetyl &[+446.96]. *B,* MS2 spectrum of Calm1-N-acetyl&acetyl, *C,* MS2 spectrum of Calm1-N-acetyl&acetyl &[+446.96]. *D,*  MS2 spectrum of Calm1-N-acetyl-2acetyl. Calm1[N-acetyl&acetyl at 1120.393 m/z existed in the same MS1 spectrum with Calm1[N-acetyl&acetyl &446.956] (at 1150.27, **Fig. S7A**). From the accurate mass in the MS1 spectrum, an extra mass of 446.959 Da could confirmed matched to 1150.270 *m/z* (15+), yet the MS2 spectrum was insufficient to identify the PTM/potential noncovalent adduct (**Fig. S7C**). Similar challenge of PTM localization was seen for Calm1-N-acetyl-2acetyl. Thus only total PTM composition was reported for these proteoforms.


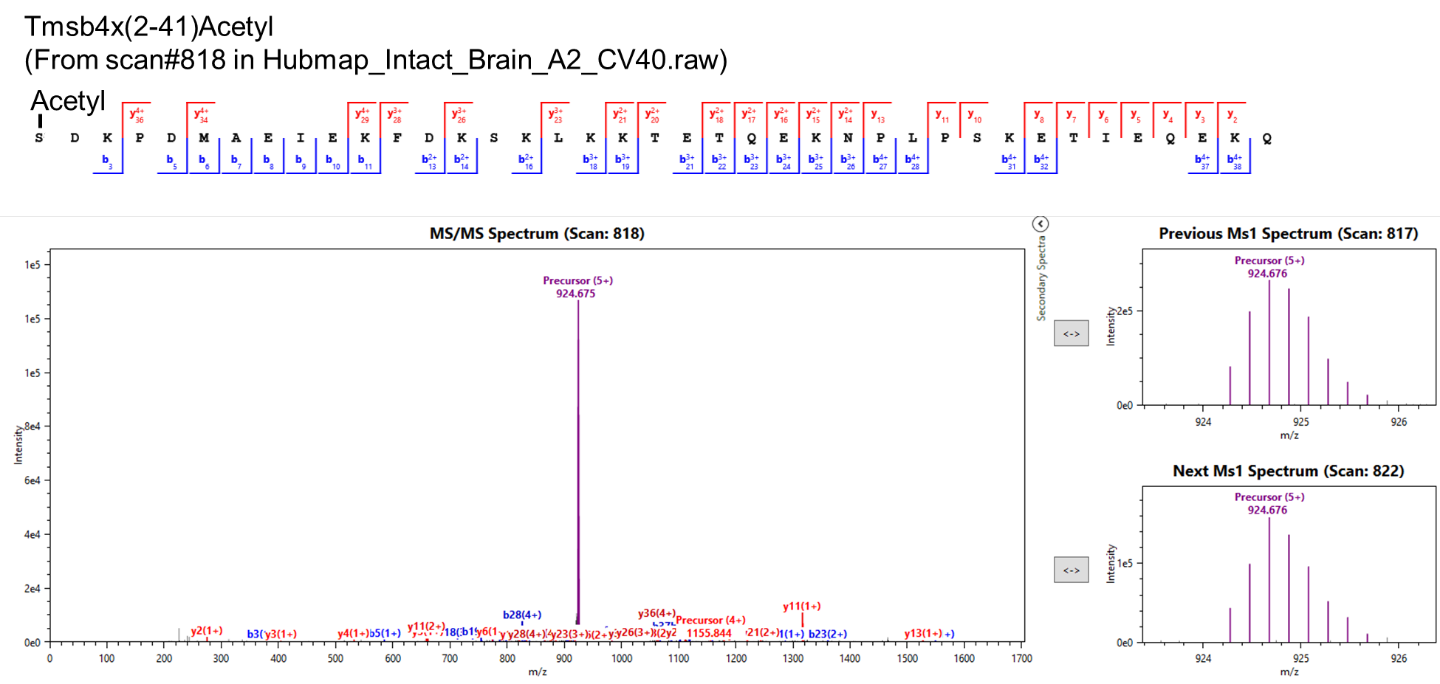

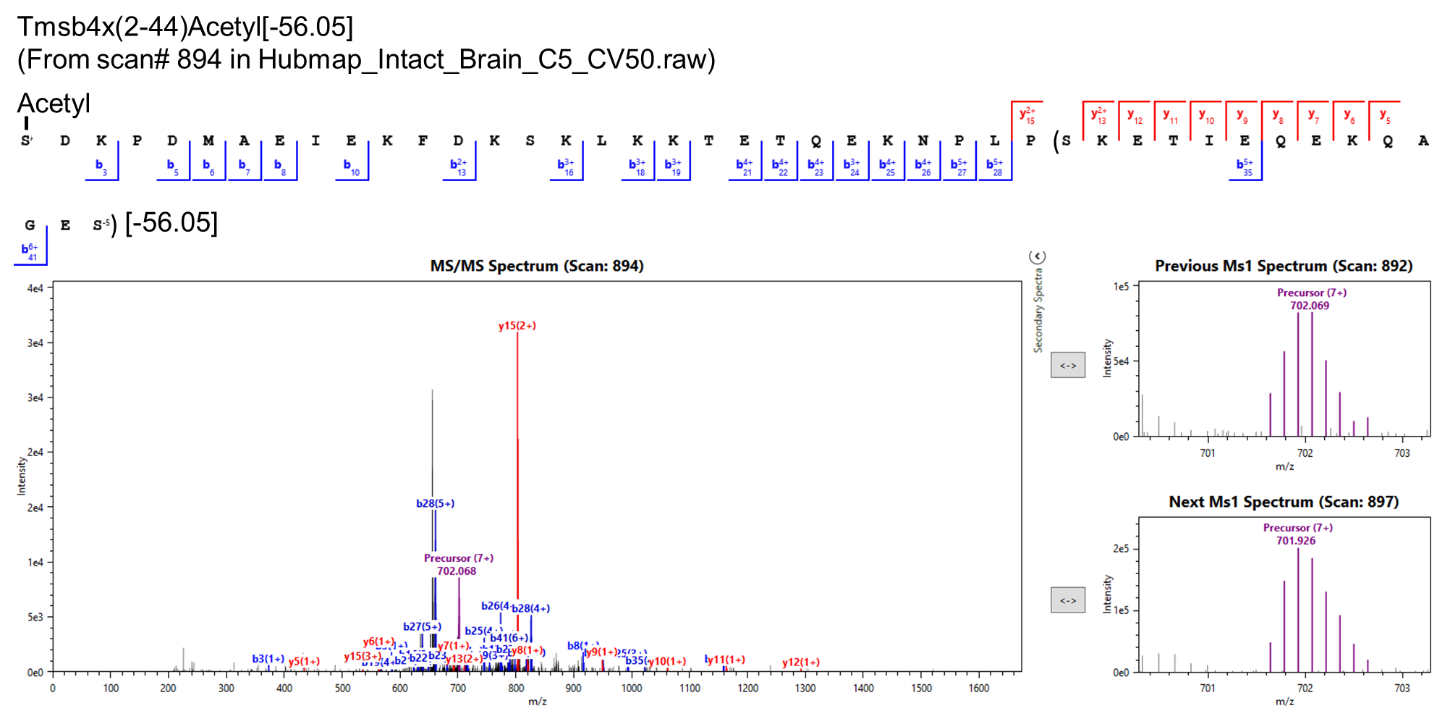


**Supporting Figure S8. Representative spectra from proteoforms of Tmsb4x.** Annotated spectra for Tmsbx4 N-acetyl (top) and N-acetyl-[-56.0498] (bottom) proteoforms. Both spectra showed good sequence coverage, large number of matched fragments, and good signal for precursor ions. For the proteoform with mass shift of -56.05 at the C-terminus, truncation of residues alone cannot explain the observed mass difference.


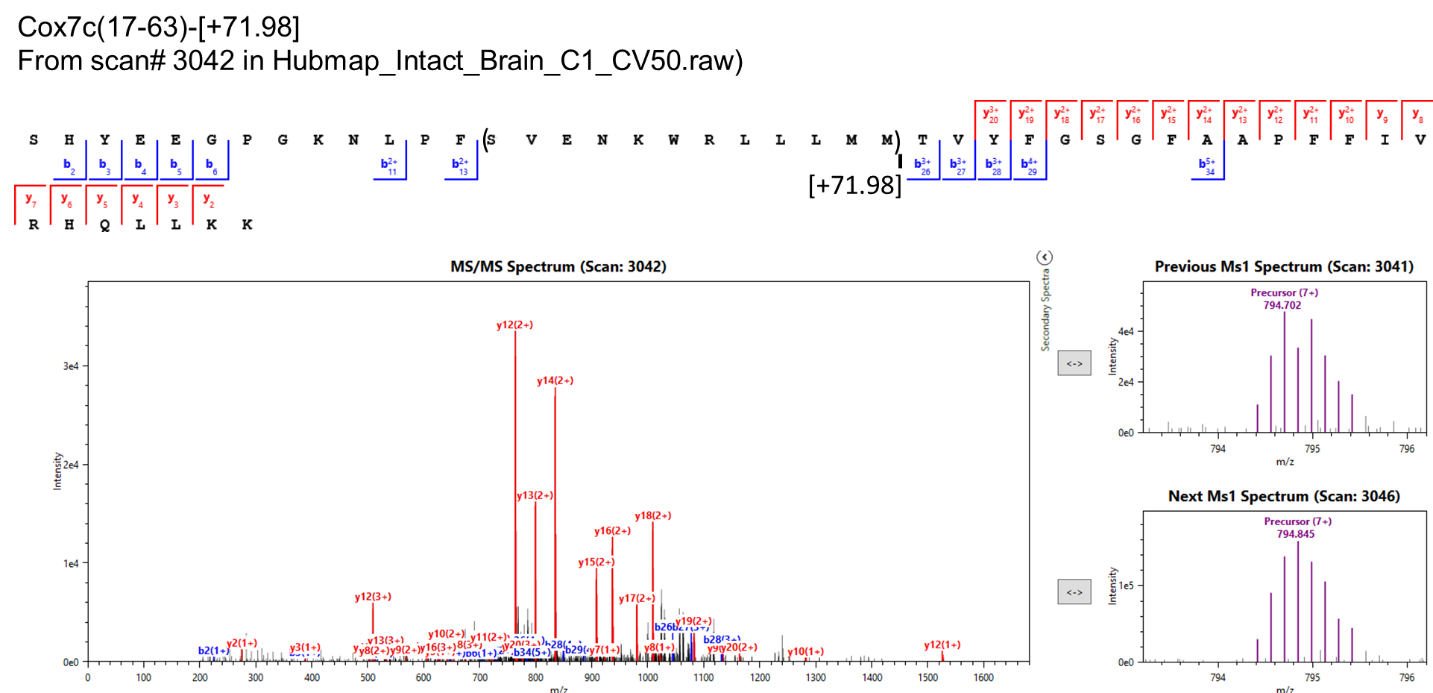

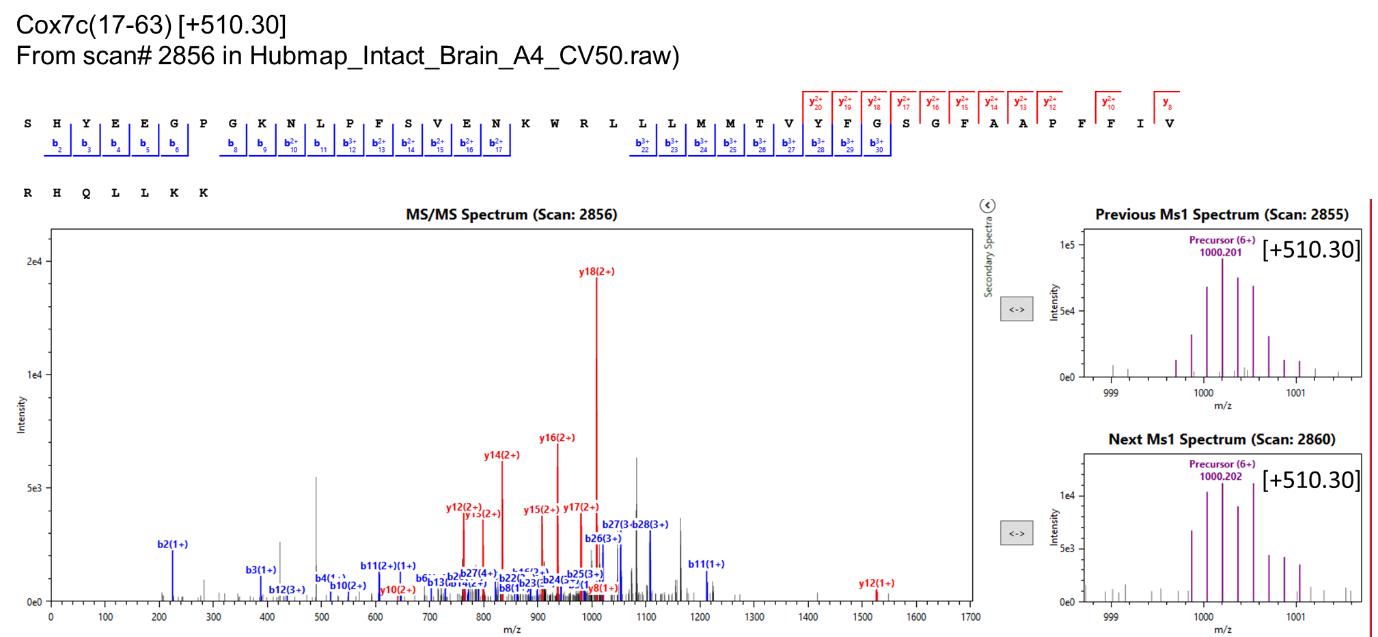


**Supporting Figure S9. Representative spectra from proteoforms of Cox7c.** Annotated spectra for two Cox7c proteoforms with unknown mass shifts. Both spectra had high sequence coverage and good isotope fit for precursor matches. Mass shift of 71.98 Da may represent a combination of PTMs in the middle of the protein. Mass shift of 510.30 Da likely represents a noncovalent adduct or a labile PTM. The fragment spectra matched well to the unmodified protein, but the precursor ion contained an extra mass of 510.30 Da, implying the PTM was lost during fragmentation.


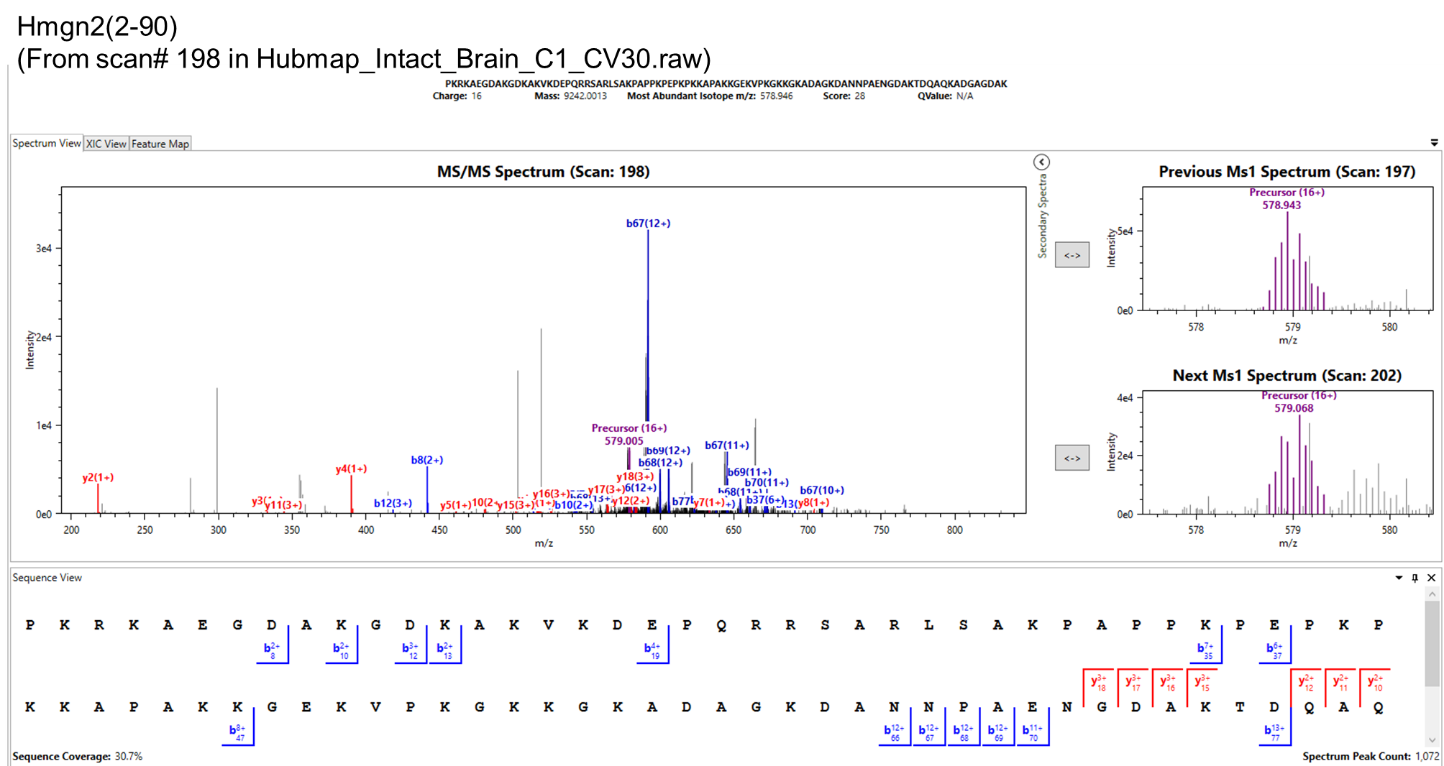


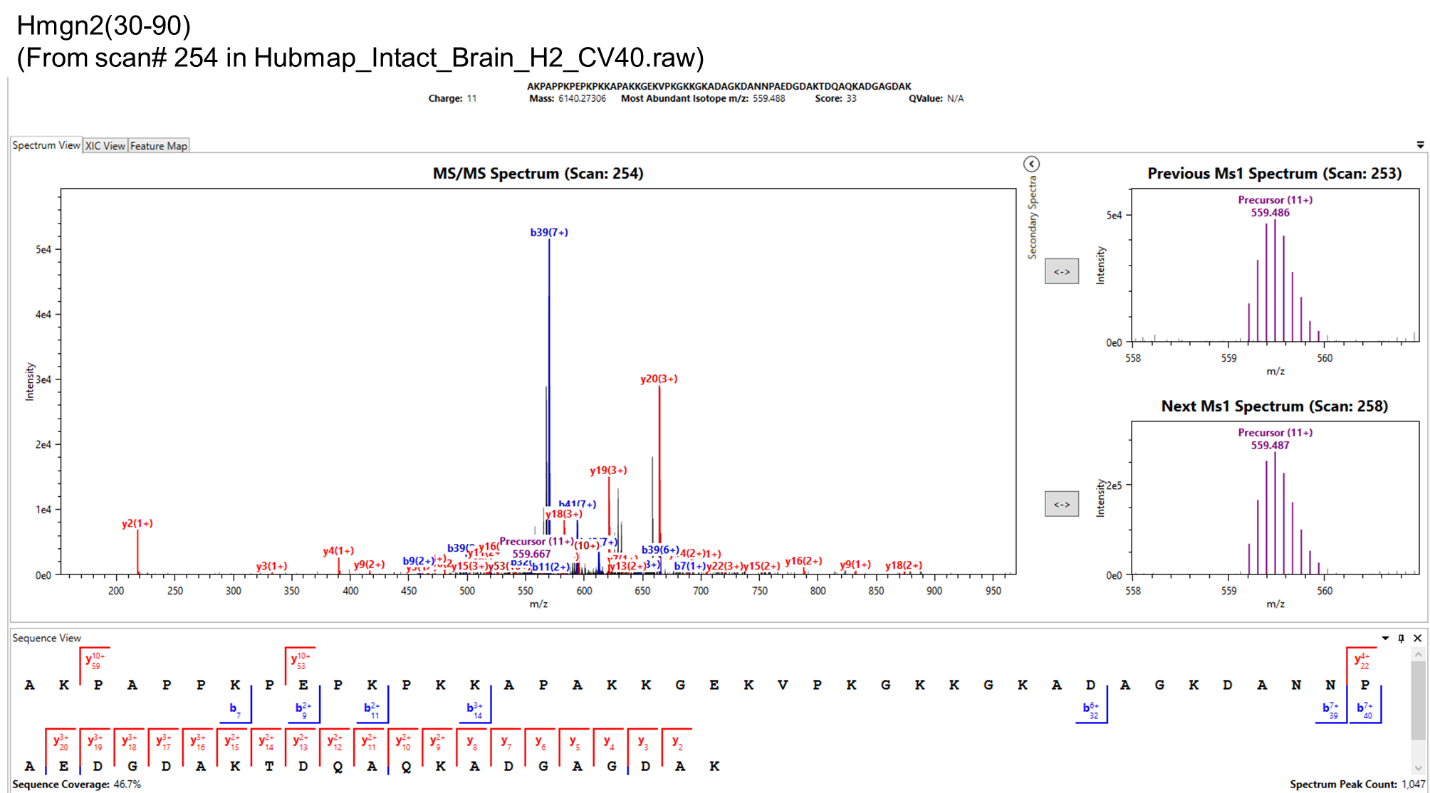


**Supporting Figure S10. Representative spectra from proteoforms of Hmgn2.** Annotated spectra for Hmgn2(2-90) (top) and Hmgn2(30-90) (bottom) proteoforms. Both spectra showed good sequence coverage, large number of matched fragments, and good signal for precursor ions.

**
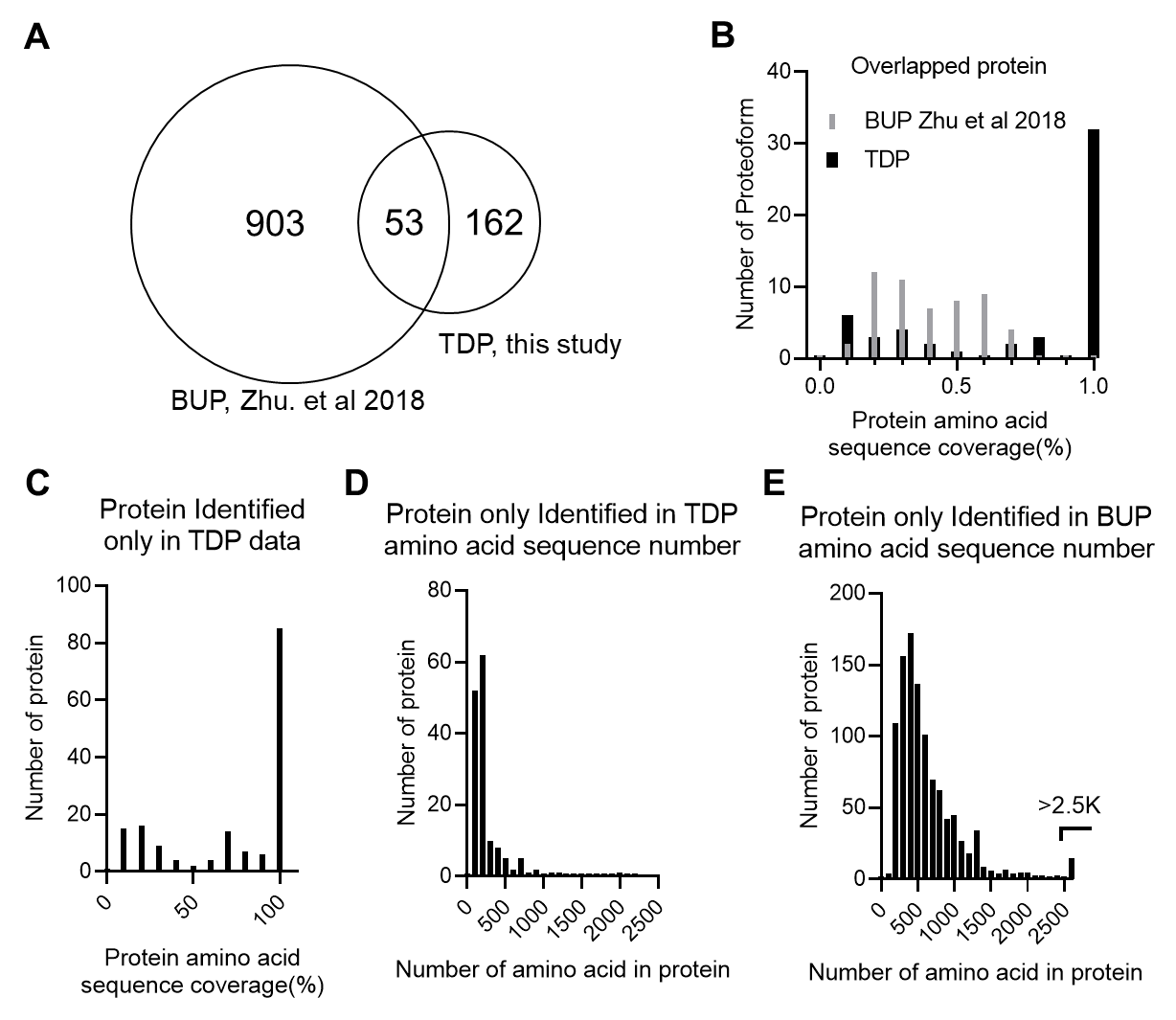
**

**Supporting Figure S11. Comparison of our TDP study with a recent BUP study of rat brain tissue with similar nanoPOTS platforms.** *A,* Overlap of protein identifications. There were 956 proteins identified in the previous BUP data and only 53 proteins were shared in this study. *B,* In the 53 overlapped proteins, 32 proteins with their proteoforms had over 90% coverage over the UniProt full sequences, indicating near complete characterization (not with special consideration for signaling peptides, etc). In contrast, most proteins showed < 50% peptide coverage over full amino acid sequences in BUP. *C,* Among the 162 unique protein identification in TDP, 50% had >90% coverage, suggesting there were (near)-full-length proteoforms and not small degradation products. *D,* Amino acid length of full sequences in UniProt for all detected proteins in TDP data. A majority of them were within the detectable range of our TDP method (<30 kDa). Some high mass proteins were also detected, which should be attributed from low mass fragments. *E,* Amino acid length of full sequences in UniProt for protein only identified in BUP data. Most of proteins are around 200~ 1000 amino acids. Overall the high-mass proteins were underrepresented in TDP.
